# Single-molecule imaging of stochastic interactions that drive dynein activation and cargo movement in cells

**DOI:** 10.1083/jcb.202210026

**Published:** 2024-01-19

**Authors:** Nireekshit Addanki Tirumala, Gregory Michael Ian Redpath, Sarah Viktoria Skerhut, Pritha Dolai, Natasha Kapoor-Kaushik, Nicholas Ariotti, K. Vijay Kumar, Vaishnavi Ananthanarayanan

**Affiliations:** 1 Centre for BioSystems Science and Engineering, Indian Institute of Science, Bengaluru, India; 2 EMBL Australia Node in Single Molecule Science, Department of Molecular MedicineSchool of Biomedical Sciences, University of New South Wales, Sydney, Australia; 3 International Centre for Theoretical Sciences, Tata Institute of Fundamental Research, Bengaluru, India; 4 Electron Microscopy Unit, University of New South Wales, Sydney, Australia

## Abstract

Cytoplasmic dynein 1 (dynein) is the primary minus end–directed motor protein in most eukaryotic cells. Dynein remains in an inactive conformation until the formation of a tripartite complex comprising dynein, its regulator dynactin, and a cargo adaptor. How this process of dynein activation occurs is unclear since it entails the formation of a three-protein complex inside the crowded environs of a cell. Here, we employed live-cell, single-molecule imaging to visualize and track fluorescently tagged dynein. First, we observed that only ∼30% of dynein molecules that bound to the microtubule (MT) engaged in minus end–directed movement, and that too for a short duration of ∼0.6 s. Next, using high-resolution imaging in live and fixed cells and using correlative light and electron microscopy, we discovered that dynactin and endosomal cargo remained in proximity to each other and to MTs. We then employed two-color imaging to visualize cargo movement effected by single motor binding. Finally, we performed long-term imaging to show that short movements are sufficient to drive cargo to the perinuclear region of the cell. Taken together, we discovered a search mechanism that is facilitated by dynein’s frequent MT binding–unbinding kinetics: (i) in a futile event when dynein does not encounter cargo anchored in proximity to the MT, dynein dissociates and diffuses into the cytoplasm, (ii) when dynein encounters cargo and dynactin upon MT binding, it moves cargo in a short run. Several of these short runs are undertaken in succession for long-range directed movement. In conclusion, we demonstrate that dynein activation and cargo capture are coupled in a step that relies on the reduction of dimensionality to enable minus end–directed transport in cellulo and that complex cargo behavior emerges from stochastic motor–cargo interactions.

## Teaser

Using live-cell single-molecule imaging, Tirumala and Redpath et al. show that the motor protein cytoplasmic dynein is activated in a stochastic manner when it encounters preformed dynactin–cargo complexes on microtubules. The resulting cargo movement in short bursts cumulatively results in long-range transport into late endosomal compartments.

## Introduction

The dynein family of motor proteins comprises axonemal and cytoplasmic dyneins. While cytoplasmic dynein 2 plays a critical role in intraflagellar transport (Höök and Vallee, 2006), cytoplasmic dynein 1 (dynein henceforth) is responsible for force production and minus end–directed movement of a variety of cargo in cells containing microtubules (MTs) (Allan, 2011). Dynein is a large complex of homodimers consisting of 500 kD heavy chains and other accessory proteins including light chains, light intermediate chains, and intermediate chains, which mediate dimerization of dynein and thereby its processivity. The activity of motor proteins is typically regulated: several kinesins assume an autoinhibited conformation until attachment to cargo (Verhey and Hammond, 2009); dynein was first found to be regulated for its processivity by the multisubunit complex, dynactin (King and Schroer, 2000). More recent studies have additionally implicated cargo adaptors—which link dynein to a multitude of cargo—in the activation of dynein (Vallee et al., 2012; Cianfrocco et al., 2015; Ananthanarayanan, 2016; Canty and Yildiz, 2020). Dynactin is a large multisubunit complex that was first identified as an activator of minus end–directed motility of vesicles (Gill et al., 1991). Further research indicated that an intact dynactin complex was necessary for dynein’s function and that dynactin could interact with MTs via its p150 subunit (Quintyne and Schroer, 2002; Valetti et al., 1999). The N-terminal CAP-Gly domain on p150 was also found to be able to interact with growing MT plus ends via EB1/CLIP-170 pathway (Vaughan et al., 1999; Watson and Stephens, 2006) and influence intracellular transport (Vaughan et al., 2002). However, the interaction between dynein and dynactin, as probed from coimmunoprecipitation assays, was observed to be weak, and overexpressing the N-terminal (cytoplasmic) fragment of the cargo adaptor BicD2 was found to significantly increase dynein–dynactin interaction (Splinter et al., 2012).

Cargo adaptors are proteins that link membranous cargo to the motor (Hoogenraad and Akhmanova, 2016), with specific cargo adaptors being employed for different types of cargo (Reck-Peterson et al., 2018; Olenick and Holzbaur, 2019). Recent single-molecule in vitro research has established that formation of the dynein–dynactin–cargo adaptor (DDC) complex is essential for processive motion (McKenney et al., 2014; Schlager et al., 2014). Cryo-EM studies later revealed that the formation of the DDC complex relieved the autoinhibition of dynein and reoriented the motor for processive movement (Torisawa et al., 2014; Zhang et al., 2017; Chowdhury et al., 2015). The DDC complex enhances the force produced by single dynein motors from 1 pN to about 4.3 pN (Belyy et al., 2016). Thus, our current understanding suggests that formation of the DDC complex is essential in dynein-driven transport. While it is clear that the tripartite complex formation is an essential first step in the activation of the dynein motor, it is unknown how this process occurs in a living cell, amid its crowded environs and the independent dynamics of each component of the tripartite complex. Here, we employ several strategies including single-molecule imaging, correlative light and electron microscopy (CLEM), and high-resolution fluorescence microscopy to propose the following mechanism for the formation of the tripartite complex and, therefore, activation of dynein: two of the three members of the tripartite complex, dynactin and the cargo adaptor (along with the cargo), remain associated and positioned along MTs; single dynein molecules stochastically bind these dynactin–adaptor complexes on the MT to complete the tripartite complex and initiate cargo movement in a short run. Multiple bouts of these short runs then result in long-range cargo transport.

## Results

### Visualization of single molecules of dynein in cells

**Figure S1.**
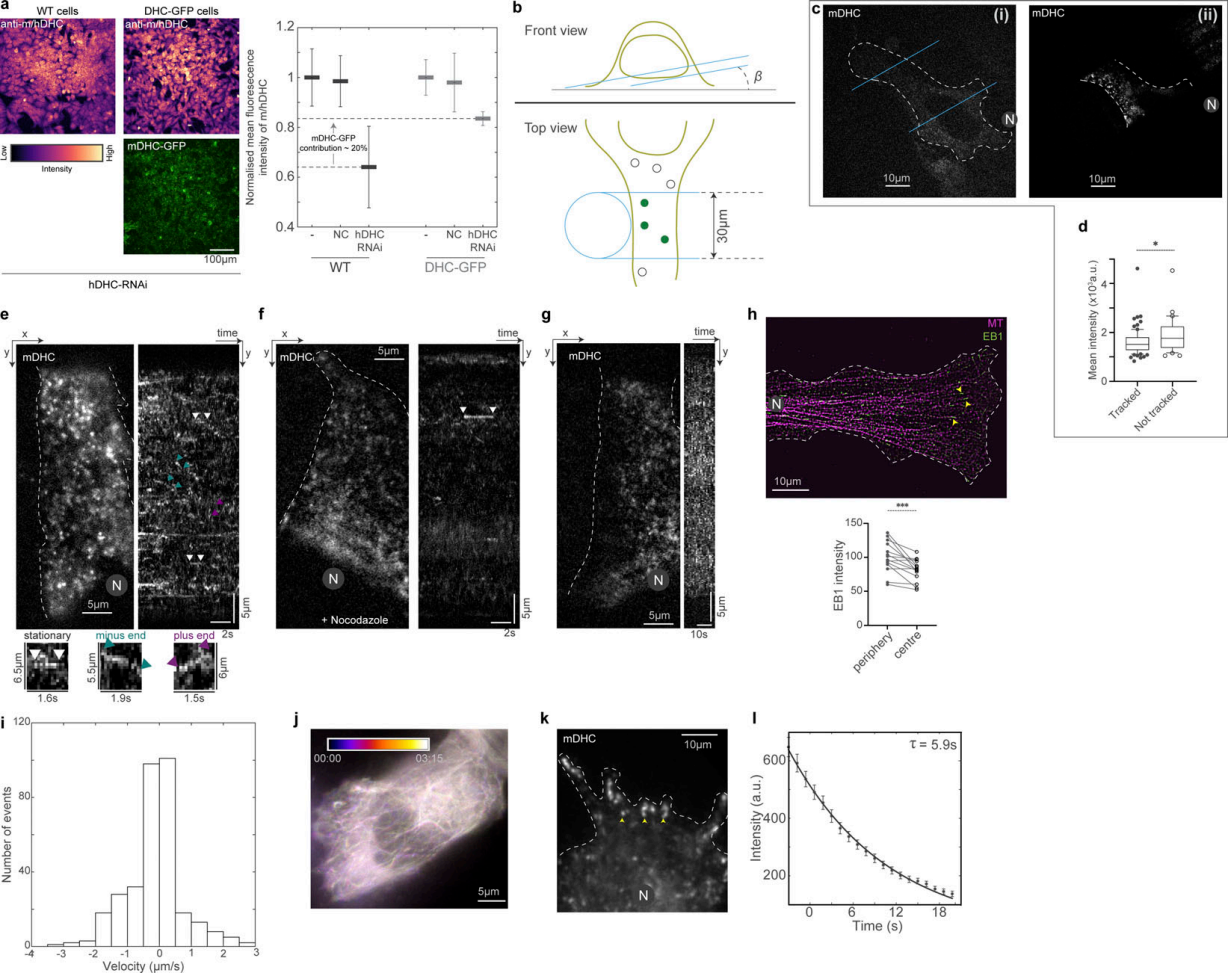
**Visualization of single molecules of dynein using HILO microscopy. (a)** Left: Confocal microscopy images (with a 20× objective) of anti-m/hDHC (rabbit polyclonal #PA5-68173 primary and donkey anti-rabbit A555 #A32794 secondary) in WT (left) and mDHC-GFP cells (right); the images are represented in the color map indicated on the bottom left and the GFP channel is provided on the bottom right. Right: Plot of mean fluorescence intensity of the anti-m/hDHC in WT and mDHC-GFP cells normalized to the respective no treatment (“−”) values (*N* = 3 independent experiments, with eight fields imaged per condition; error bars represent SEM). Following hDHC RNAi, WT cells showed normalized anti-m/hDHC intensity of 0.64 whereas DHC-GFP cells had 0.84, indicating that mDHC-GFP constitutes 20% of the dynein population in mDHC-GFP cells. **(b)** HILO microscopy setup used to visualize dynein molecules in HeLa cells. Depending on the morphology of the cell, the angle *β* was adjusted such that fluorescent spots of dynein were visible (top, “Front view”). The illumination diameter was kept constant at 30 µm using a field stop (bottom, “Top view”). This resulted in a beam thickness of ∼4.6 µm (calculated according to Tokunaga et al. [2008]). **(c)** (i) Spinning disk confocal microscopy image of a HeLa cell expressing mDHC-GFP and (ii) HILO microscopy image of the same cell that is partially illuminated in the HILO microscopy setup. The blue lines represent the orientation of the incident laser beam. In the HILO microscopy image, individual fluorescent spots are visible. **(d)** Box plot for comparison of mean intensities of cells where single-molecule events were visible (“Tracked”), and those where they were not (“Not tracked”). Data are from *n* >30 cells, *n* = 3 independent experiments; asterisk indicates P < 0.05, Mann-Whitney Test). **(e)** HILO microscopy image (left) from a 10-s-long time-lapse video of mDHC-GFP in HeLa cells and the corresponding kymograph (right). Representative stationary, minus end–directed, and plus end–directed events are indicated with the white, teal, and magenta arrowheads, respectively, in the kymograph and in the insets below. **(f)** HILO microscopy image (left) from a 10-s-long time-lapse of mDHC-GFP in cells treated with 10 µM nocodazole to depolymerize the MTs and the corresponding kymograph (right). The kymograph shows few binding events compared to the cell shown in e, indicating that dynein molecules were stochastically binding to the MTs from the cytosol. **(g)** HILO microscopy image (left) from a 20-s-long 1 fps time-lapse video of mDHC-GFP in HeLa cells and the corresponding kymograph (right). There are no distinct traces in the kymograph, indicating that the short traces shown in e are not artifacts and dynein molecules do not interact with the MTs for a long duration. **(h)** Immunofluorescence images of MT (magenta) and EB1 (green) obtained using spinning disk microscopy + SRRF. In these cells with a large aspect ratio, a majority of the MTs are plus end out (pointed by yellow arrowheads). While there might be misoriented MTs or short MTs oriented with their plus ends toward the cell center, these are likely a minority, given the higher intensity of EB1 at the cell periphery compared to the center. In all analyses, movement toward the nucleus was considered as minus end–directed transport and movement away from the nucleus as plus end directed. The plot on the bottom represents quantification of EB1 intensity at the cell periphery (farthest ends of cells covering a quarter of the cell area) and cell center (region close to the nucleus covering a quarter of the cell area; note that in our HILO movies, half a cell spanning the nucleus to the cell tip is typically visible). Data from *n* = 14 cells, *n* = 1 independent; asterisk represents P < 0.05, Wilcoxon test for paired data. **(i)** Histogram of velocities of mDHC-GFP in HeLa cells (data from Fig. 1 e). **(j)** Temporally color-coded projection of mCherry-tubulin in a 3 min 15 s video obtained using HILO microscopy with the same settings employed for single-molecule dynein imaging. The fact that the MT signal from 00:00 and 03:15 overlap significantly indicates that the MT position and dynamics do not typically vary during a typical single-molecule time-lapse movie, which is ∼10 s. Moreover, a significant length (∼20 µm) of the MT network was visible in HILO microscopy images, indicating that dynein molecules moving on MTs over long distances could be visualized and tracked. **(k)** HILO microscopy image from a time-lapse video of mDHC-GFP in a cell expressing high levels of mDHC-GFP. Clusters of dynein that are likely at the MT plus end are indicated by the yellow arrowheads. **(l)** An exponential decay fit to the intensity versus time plot of spots similar to those indicated with yellow arrowheads in k. λ = 0.17 was obtained to give the time constant 1/λ = 5.9 s. This time constant represents the average time required for a dynein molecule to bleach under our imaging conditions. This value is an order of magnitude higher than the average residence time of dynein on MTs (∼0.59 s). Data from n = 119 spots, *N* = 1 independent experiment with 25 cells. Error bars represent SEM. In c, e–h, and k, “N” marks the location/direction of the nucleus.

To probe the kinetics of dynein in living cells, we employed HeLa cells stably expressing mouse dynein heavy chain (DYNC1H1) tagged with GFP (mDHC-GFP [Poser et al., 2008]). These bacterial artificial chromosome (BAC) transgenic cell lines have been previously characterized to express mDHC-GFP at levels comparable with the endogenous protein (Poser et al., 2008; Splinter et al., 2012). We further confirmed that the cells we used in our experiments expressed low levels of mDHC-GFP, about 20% over endogenous DHC levels (Fig. S1 a). To visualize mDHC-GFP, we adapted highly inclined and laminated optical sheet (HILO) microscopy (Tokunaga et al., 2008; Fig. S1 b). When cells expressing low levels of mDHC-GFP were observed under a spinning disk confocal microscope, the fluorescence signal appeared cytosolic, with no discernible dynein punctae. However, when the same cells were observed using our modified HILO microscopy, distinct fluorescent spots were visible (Fig. S1, c and d).

**Figure 1.**
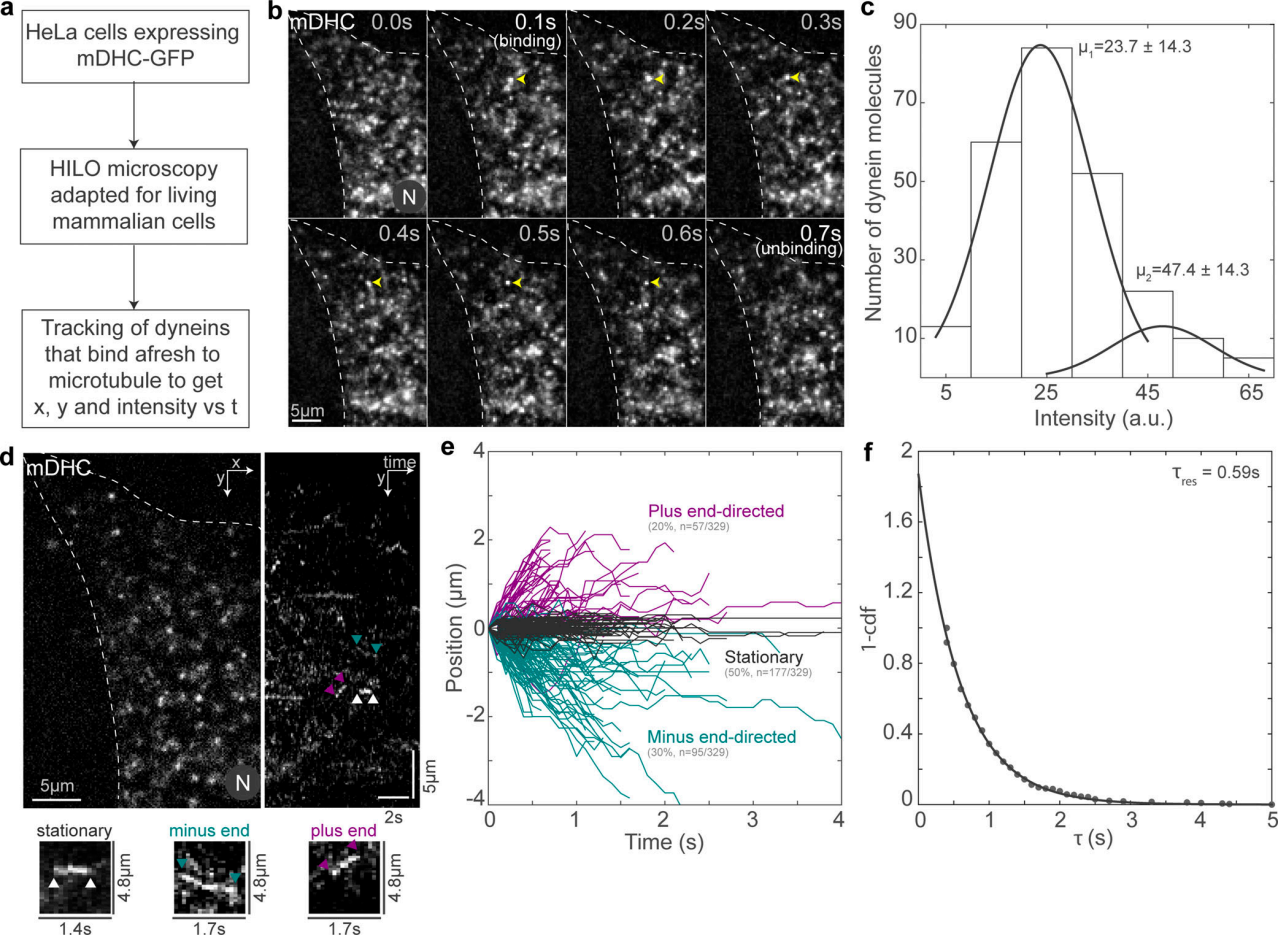
**Visualization of single molecules of dynein in living cells. (a)** Schematic of the protocol followed for the visualization of single molecules of dynein. **(b)** Montage of HILO images showing representative binding and unbinding events of a single fluorescent mDHC molecule (“[binding]” and “[unbinding]”). The single molecule is indicated with yellow arrowheads for the duration of the time it remains bound in the field of view. Time is indicated at the top right of each image in the montage. **(c)** Intensity histogram of single molecules of dynein with the Gaussian fits (gray line). The mean ± SD of the Gaussian distributions is indicated above the fits. **(d)** HILO image (left) and kymograph (right) of a cell expressing mDHC-GFP. Representative stationary, minus end–directed, and plus end–directed events are indicated with the white, teal, and magenta arrowheads, respectively, in the kymograph and in the insets below. **(e)** Plot of position versus time for the single-molecule events tracked, showing stationary events (gray), minus end–directed events (teal), and plus end–directed events (magenta). *n* = 329 tracks from ∼50 cells across three independent experiments. **(f)** Histogram with the residence time of dynein on the MT on the x axis and *P* (*τ*) = 1 − cumulative frequency on the y axis. The exponential fit (gray line) gave a mean residence time *τres* = ^1*/*^ $r_{off}^{D}$ ∼0.59 s. In b and d, “N” marks the location/direction of the nucleus.

We adapted our microscopy protocol to obscure dynein diffusing in the cytoplasm and to only observe dynein that resided on the MT (Ananthanarayanan and Tolić, 2015; Ananthanarayanan et al., 2013; Tirumala and Ananthanarayanan, 2023; Fig. 1 a; see Materials and methods). We observed that dynein spots appeared afresh and remained in the field of imaging for a short duration (Fig. 1 b and Video 1). We intuited that these corresponded to events where single dynein molecules, previously diffusing in the cytoplasm, bound to MTs (Fig. S1, e–g).

**Video 1.** **Dynein binding events.** HeLa cell expressing mDHC-GFP imaged using HILO microscopy. The green arrowhead points to a dynein molecule that became visible (a binding event), started moving toward the minus end of MT, and disappeared (an unbinding event). Similarly, the magenta arrowhead points to a dynein molecule that bound and moved to the MT plus ends and the white arrowhead points to a stationary dynein molecule. “N” marks the position/direction of the nucleus. Imaged at 50 fps, movie playback 50 fps. Scale bar: 5 µm. Movie related to Fig. 1.

To confirm that the appearance of fluorescent signal on the MT corresponded to binding of a single molecule of dynein to the MT, we analyzed the intensity of these fluorescent spots (Fig. 1 c). For single dynein molecules, we would expect the intensity histogram to fit to a sum of two Gaussian distributions, one corresponding to a GFP fluorescing from one DHC and the other corresponding to two GFPs fluorescing from both DHCs in the genetic background of these cells. The former primarily arose due to photobleaching of GFP during the course of imaging. Accordingly, the intensity histogram of these fluorescent spots revealed that these were most likely single dynein molecules since the intensity histogram fit best to a sum of two Gaussians, with the mean of the first Gaussian profile being half that of the second (Fig. 1 c). Additionally, given that we did not see clusters of mDHC-GFP in the cytoplasm when imaging using confocal microscopy (Fig. S1 c), it is unlikely that these fluorescent spots arriving afresh from the cytoplasm on to the MTs in our HILO imaging comprise large numbers of dyneins.

### Dynein interacts transiently with the MT

Next, to probe the behavior of dynein upon binding to MT, we analyzed the two-dimensional position (*x* and *y*) versus time (*t*) of single molecules of dynein that bound afresh from the cytoplasm to the MT (Fig. 1 d). Based on automated thresholding (see Materials and methods), we classified the tracks as stationary, minus end directed, and plus end directed. By quantifying the localization and intensity of EB1, a protein that tracks the growing plus ends of MTs, we observed that the plus ends of the MT were predominantly at the periphery in these elongated cells (Fig. S1 h). Therefore, we annotated movement of single dynein molecules toward the cell center as minus end directed and movement away as plus end directed. We observed that ∼50% of all the dynein molecules tracked (*n* = 177/329, *N* = 3 independent experiments from >50 cells) remained stationary upon MT binding, while ∼30% (*n* = 95/329) moved toward the minus end (Fig. 1 e). The remaining ∼20% moved toward the plus end, and these arose likely due to attachment of dynein to cargo being moved to the plus end by kinesins (Fig. 1 e).

The velocity measured for minus end–directed movement of single dyneins was 1.2 *±* 0.7 µm/s (mean *±* SD, Fig. S1 i), similar to values reported for mammalian dynein previously (Flores-Rodriguez et al., 2011; Zajac et al., 2013). The mean plus end velocity of dynein molecules was 1.1 ± 0.7 μm/s (mean ± SD), consistent with velocities of processive kinesin-1 in cells reported previously (Cai et al., 2007). We also confirmed that the underlying MT was stable, did not undergo sliding, and therefore did not contribute to the dynein behavior we observed (Fig. S1 j). We then measured the mean residence time (*τ*_res_) of dynein on the MTs to be 0.59 s (95% confidence interval: 0.57–0.61 s; Fig. 1 f). The unbinding rate of dynein from MT $r_{off}^{D}=1/\tau_{res}$ was 1.7 s^−1^, which is similar to the previously reported unbinding rate of single dyneins (Kunwar et al., 2011).

**Figure S2.**
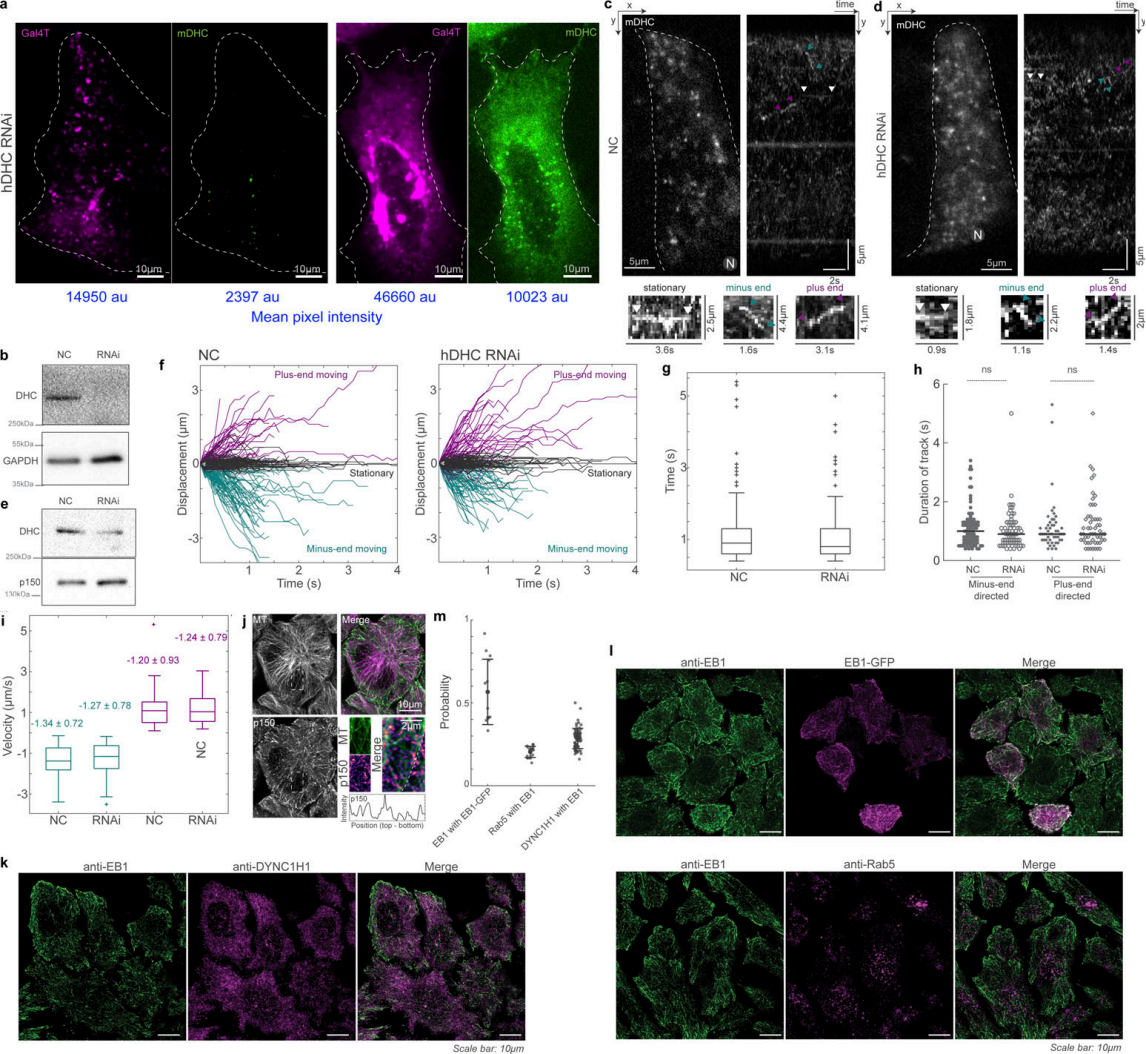
**mDHC-GFP is functional in HeLa cells depleted of endogenous DHC. (a)** Spinning disk microscopy images of live cells expressing Gal4T mCherry (magenta) and mDHC-GFP (green). The cells were treated with 25 nM siRNA against endogenous hDHC. The cell on the left had little mDHC-GFP signal and a dispersed Golgi apparatus whereas the cell on the right expressed higher levels of mDHC-GFP and had the Golgi clustered at the cell center. Quantification revealed that 100% of the cells without mDHC-GFP expression had a dispersed Golgi whereas only 24% of the cells expressing mDHC-GFP had a dispersed Golgi, indicating mDHC-GFP was functional (*n* = 34 cells from *n* = 2 independent experiments). The mean pixel intensities (in arbitrary units [au]) of the Gal4T and mDHC-GFP channels are indicated below the images in blue. **(b)** Representative western blot to verify the knock down of hDHC by RNAi in a. Quantification of the western blot confirmed that the RNAi successfully knocked down levels of hDHC by >90% (*n* = 2 independent experiments). **(c)** HILO microscopy image (left) from a 10-s-long time-lapse of mDHC-GFP in cells treated with 25 nM NC siRNA and the corresponding kymograph (right). Representative stationary, minus end–directed, and plus end–directed events are indicated with white, teal, and magenta arrowheads, respectively, in the kymograph and in the insets below. **(d)** HILO microscopy image (left) from a 10-s-long time-lapse of mDHC-GFP in cells treated with 25 nM hDHC siRNA and the corresponding kymograph (right). Representative stationary, minus end–directed, and plus end–directed events are indicated with white, teal, and magenta arrowheads, respectively, in the kymograph and in the insets below. **(e)** Representative western blot to verify the knockdown of DHC by RNAi in d. Quantification of the western blot confirmed that the RNAi successfully knocked down levels of DHC by an average of 72% (*n* = 2 independent experiments). **(f)** Comparison of displacement versus time plots of mDHC-GFP molecules in cells treated with 25 nM NC siRNA (left) and 25 nM siRNA against endogenous hDHC (right). In cells treated with 25 nM NC siRNA, 53% of the molecules remained stationary, 31% moved toward the minus ends of the MTs, and 16% moved toward the plus ends of the MTs, whereas in cells treated with 25 nM siRNA against hDHC, 52% of the molecules remained stationary, 29% moved toward the minus ends of the MTs, and 19% moved toward the plus ends of the MTs. **(g)** Box plots comparing the residence time of mDHC-GFP molecules in cells treated with 25 nM NC siRNA versus cells treated with 25 nM siRNA against endogenous hDHC indicating no significant differences. **(h)** Comparison of duration of plus end– and minus end–directed runs of mDHC-GFP in cells with NC RNAi and hDHC RNAi. “ns” represents no significant difference (Mann–Whitney *U* test). **(i)** Box plots comparing minus end–directed velocities (teal boxes) of mDHC-GFP molecules in cells treated with 25 nM NC siRNA and 25 nM siRNA against endogenous DHC, showing no significant differences. Similarly, the plus end–directed velocities (magenta boxes) were also not significantly different. The mean ± SD of velocities is indicated in the box plot. In f–i, data for NC was obtained from *n* = 254 binding events tracked from *n* = 3 independent experiments with >30 cells. Data for RNAi was obtained from *n* = 245 binding events tracked from *n* = 3 independent experiments with >30 cells. **(j)** Immunofluorescence images of MTs (top left, magenta), p150 (bottom left, green), and their merge (top right), obtained using Airyscan confocal microscopy showing enrichment of p150 at MT tips. The inset indicated with the white box is on the bottom right, with the normalized intensity profile of p150 along the MT indicated with the dashed white line, showing localization of p150 along the entire MT. Compare this image to Fig. 2 a, imaged with another p150 antibody. **(k)** Fluorescence images of anti-EB1 (left, green), anti-DYNC1H1 (magenta, center), and their merge (right) obtained using Airyscan confocal microscopy. **(l)** Fluorescence images of anti-EB1 (left, green), EB1-GFP (top center, magenta), anti-Rab5 (bottom center, magenta), and their merge (right), obtained using Airyscan confocal microscopy. **(m)** Quantification (mean *±* SD) of the co-occurrence of the different proteins imaged in k and l. Note that “EB1 with EB1-GFP” is a positive control and represents the maximum co-occurrence that is quantifiable in cells; “Rab5 with EB1” is a negative control, since EB1 and Rab5 do not typically interact. Each dot represents an individual cell analyzed (*n* = 13–74 cells from *n* = 2–3 independent experiments); error bars represent SD. In c and d, “N” marks the location/direction of the nucleus. Source data are available for this figure: SourceData FS2.

We verified that this short residence time of dynein on MTs was a true representation of the duration of time that dynein remained attached to the MT and not convolved by GFP’s photobleaching time (Fig. S1, k and l). Further, by knocking down endogenous HeLa DYNC1H1 (hDHC), we verified that our observations were not an artifact of expression of mDHC-GFP in this background (Fig. S2, a–i). In cells with no discernible mDHC-GFP, knockdown of hDHC led to dispersion of the Golgi marker GalT, while cells expressing low levels of mDHC-GFP (similar to those chosen for single-molecule imaging) had no effect on Golgi dispersion, showing that mDHC-GFP in these cells functions as expected (Fig. S2, a and b). We performed single-molecule analysis of dynein in control (Fig. S2 c) or siRNA (Fig. S2 d) treated mDHC-GFP HeLa cells. We verified the knockdown of hDHC by western blot (Fig. S2 e) and observed no difference in mDHC-GFP behavior, namely proportions of stationary, plus end–, and minus end–directed movement (Fig. S2 f), residence time on MTs (Fig. S2, g and h), and velocities (Fig. S2 i). Taken together, mDHC-GFP behaves identically in the presence or absence of hDHC, functionally rescues hDHC depletion, does not induce aberrant dynein behavior, and thus represents a good model to test dynein function in cellulo. To the best of our knowledge, these are the first observations of single molecules of dynein in mammalian cells and indicate that dynein likely exists in an inactive state inside the cell, similar to reports from in vitro studies (Splinter et al., 2012; McKenney et al., 2014; Schlager et al., 2014; Zhang et al., 2017).

### Dynactin localizes to the entire MT lattice

Next, we aimed to visualize the dynamics of dynactin, the second player in the tripartite complex. Dynactin was first identified as a complex that was required for dynein-driven motility of vesicles in vitro (Gill et al., 1991). Several recent pieces of research have identified dynactin as an essential part of the active dynein complex (King and Schroer, 2000; Chowdhury et al., 2015; Urnavicius et al., 2015). Dynactin is a multisubunit complex that binds to MTs independently of dynein via its N-terminal p150 subunit (Culver-Hanlon et al., 2006). However, dynactin interacts with dynein poorly in the absence of cargo adaptor (Splinter et al., 2012; McKenney et al., 2014; Schlager et al., 2014).

**Figure 2.**
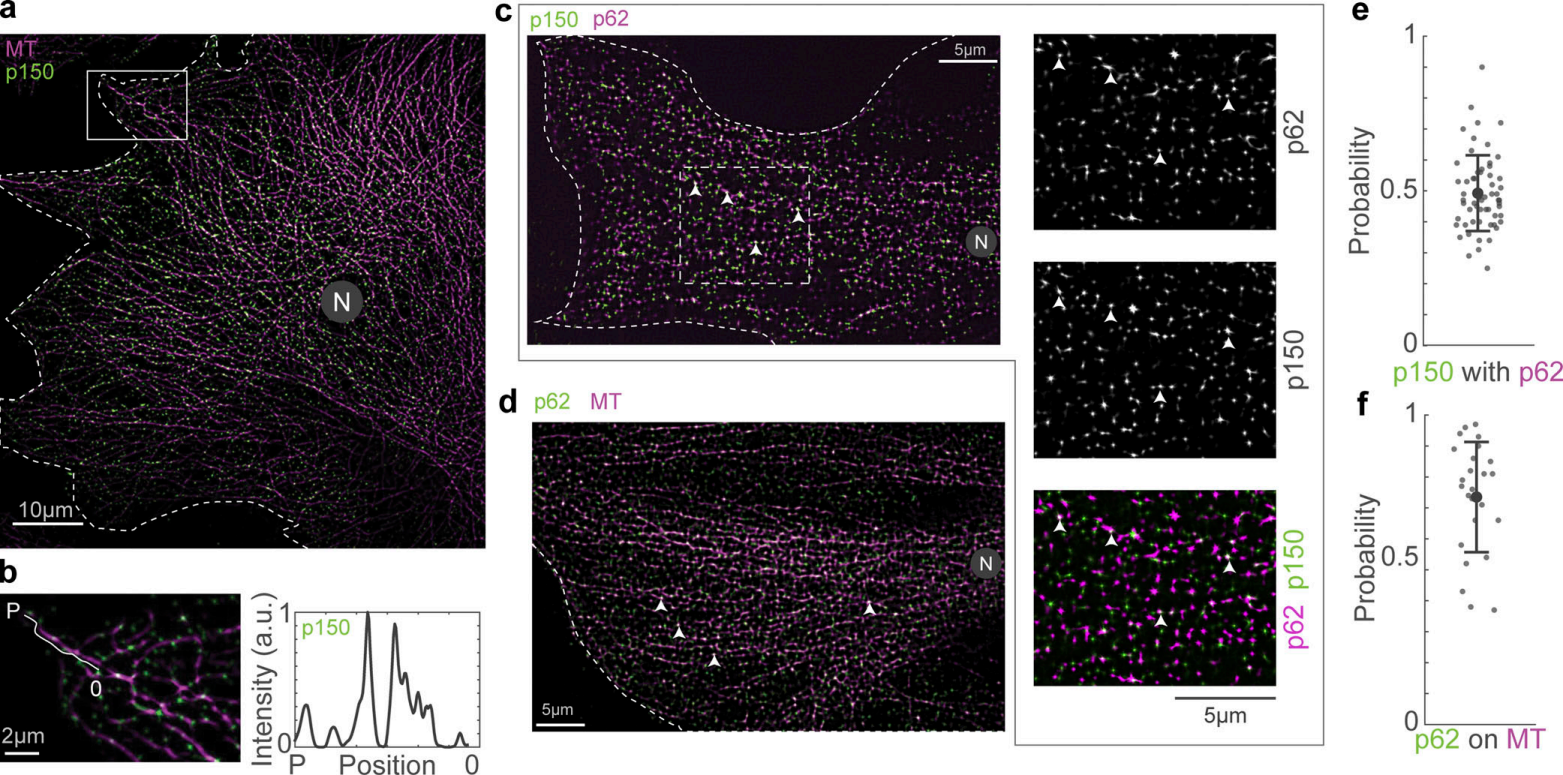
**The dynactin complex binds along the entire length of the MT. (a)** Immunofluorescence image of MT (magenta) and p150 (green) obtained using spinning disk microscopy + SRRF. **(b)** Enlarged view of the area marked with the white rectangle in a and the line profile of p150 intensity along the length of a representative MT trace (white line is representative MT trace used for the line profile) from the plus end (“P”) to ∼6 µm from the plus end of the MT (“0”). **(c)** Immunofluorescence images of p62 (green) and p150 (magenta) obtained using spinning disk microscopy + SRRF. Enlarged views of the area marked with the white rectangle are shown as individual channel images and their merge to the right of the image. The white arrowheads indicate representative p150 spots that also contain p62. **(d)** Immunofluorescence image of MT (magenta) and p62 (green) obtained using spinning disk microscopy + SRRF. The white arrowheads indicate representative p62 spots that occur on the MT. **(e)** Plot of the probability of co-occurrence of p62 with p150, indicating a high likelihood of presence of the entire complex at a p150 spot. *n* = 59 cells across two independent experiments. **(f)** Plot of the probability of co-occurrence of p62 on the MT, which points to a high likelihood for the presence of the entire dynactin complex on the MT. *n* = 25 cells across one independent experiment. In a, c, and d, “N” marks the location/direction of the nucleus. Error bars in e and f represent SD.

**Figure S3.**
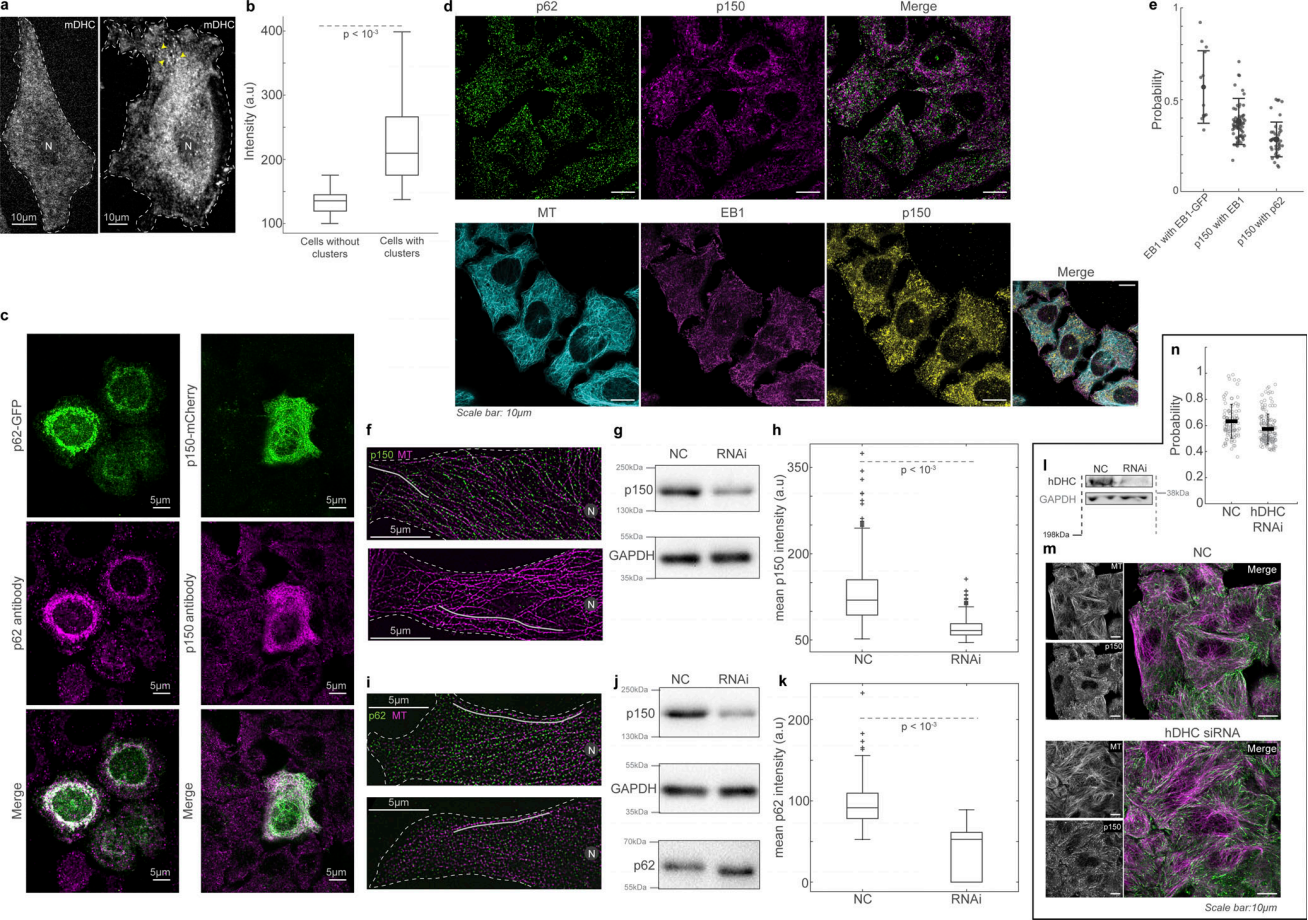
**Dynactin’s association with MTs. (a)** Spinning disk microscopy images of live cells expressing mDHC-GFP, with no visible dynein clusters (left) and with distinct dynein clusters (yellow arrowheads) (right). **(b)** Box plots comparing the average intensities between cells with and without dynein clusters, showing that cells with higher total mDHC intensities exhibited clusters. Therefore, clusters of dynein were likely a result of high overexpression of mDHC. (*n* = 25 cells per condition from *n* = 1 independent experiment). **(c)** Left: Fluorescence images of p62-GFP (top, green), anti-p62 (magenta, middle), and their merge (bottom). Right: Fluorescence images of p150-GFP (green, top), anti-p150 (Invitrogen antibody, magenta, middle), and their merge (bottom). These images indicate that the antibodies employed to detect p62 and p150 in this study are specific. **(d)** Top: Immunofluorescence images of p62 (left, green), p150 (center, magenta), and their merge (right). Bottom: Immunofluorescence images of MT (left, cyan), EB1 (second from left, magenta), p150 (third from left, yellow), and their merge (right). All images were obtained using Airyscan confocal microscopy. **(e)** Quantification (mean *±* SD) of the co-occurrence of the different proteins imaged in d. Note that “EB1 with EB1-GFP” has been reused from Fig. S2 k. Each dot represents an individual cell analyzed (*n* = 45–74 cells from *n* = 2–3 independent experiments). Error bars represent SD. All images were obtained using Airyscan confocal microscopy. **(f)** Immunofluorescence images of MT (magenta) and p150 (green) obtained using spinning disk microscopy + SRRF on cells treated with 35 nM NC siRNA (top) and 35 nM p150 siRNA (bottom). **(g)** Representative western blot to verify the knockdown of p150 by RNAi. Quantification of the western blot confirmed that the RNAi successfully knocked down levels of p150 by an average of 60% (two independent experiments). **(h)** Box plots comparing the mean intensity of p150 along MTs (representative ROI shown as a gray line in f) between cells treated with NC and p150 siRNA, showing that p150 knockdown reduces p150 levels along MTs. For each condition, 500 MT segments of length 15 *±* 6 µm (mean *±* SD) were analyzed from *n* = 50 cells from *n* = 2 independent experiments. **(i)** Immunofluorescence images of MT (magenta) and p62 (green) obtained using spinning disk microscopy + SRRF on cells treated with 35 nM NC siRNA (top) and 35 nM p150 siRNA (bottom). **(j)** Representative western blot to verify the knockdown of p150 by RNAi. Quantification of the western blot confirmed that the RNAi successfully knocked down levels of p150 by an average of 60% (*n* = 2 independent experiments). Moreover, there were no significant differences in the levels of p62 in cells treated with NC and p150 siRNA. **(k)** Box plots comparing the mean intensity of p62 along MTs (representative ROI shown as gray line in i) between cells treated with NC and p150 siRNA showing that p150 knockdown reduces p62 levels along MTs. For NC, ∼190 MT segments of length 15 *±* 6 µm (mean *±* SD) were analyzed from *n* = 51 cells from *N* = 2 independent experiments. For p150 RNAi, ∼100 MT segments of length 17 *±* 9 µm (mean *±* SD) were analyzed from *n* = 52 cells from *n* = 2 independent experiments.  **(l)** Representative western blot to verify the knockdown of hDHC by RNAi. Quantification of western blot confirmed that the RNAi successfully knocked down levels of endogenous hDHC by an average of 74% (*n* = 3 independent experiments). **(m)** Immunofluorescence images of p150 and MTs (left), and their merge (green and magenta, respectively, right) in NC (top) and hDHC siRNA cells (bottom), obtained using Airyscan confocal microscopy. **(n)** Quantification of the proportion of p150 that colocalized with MTs in NC and hDHC siRNA cells (*n* > 104 cells from *n* = 3 independent experiments, error bars represent SEM). In the NC cells, 63.2 ± 14.0% of p150 signal was on the MTs, whereas hDHC siRNA cells showed a small yet significant reduction (P < 0.01, Kruskal–Wallis test) to 57.3 ± 11.5% of p150 signal on MTs. This indicates that loss of DHC reduced p150’s loading to MTs only by a small extent of ∼6%. In a, f, and i, “N” marks the location/direction of the nucleus. Source data are available for this figure: SourceData FS3.

Dynactin has been described to cluster at growing MT plus ends via its p150 subunit; the p150 subunit binds the CLIP-170, which tracks the growing plus ends of MTs in an EB1-dependent fashion (Watson and Stephens, 2006). MT plus ends thus decorated with dynactin also accumulated dynein at these sites, and evidence suggested that cargo transport was initiated when these MT plus ends contacted intracellular cargo (Vaughan et al., 2002; Moughamian et al., 2013). However, MT plus end–mediated initiation of dynein-driven transport appears to vary with cell type and context (Watson and Stephens, 2006; Kim et al., 2007; Tirumala and Ananthanarayanan, 2020). Therefore, using spinning disk microscopy in combination with super-resolution radial fluctuations (SRRF; Gustafsson et al., 2016), we first quantified the localization of p150 (Fig. 2 a). Our high-resolution images revealed that while p150 was enriched at MT plus ends (Fig. S2 j, using an antibody whose epitope is in p150’s N-terminal MT-binding domain), it was also bound along the entire length of the MT lattice (Fig. S2 j and Fig. 2 b, using an antibody whose epitope is in p150’s C-terminus). Further, by visualizing hDHC along with EB1 using immunofluorescence (Fig. S2, k–m) and quantifying the intensities of mDHC-GFP expressed in our cells, we found that the significant MT plus end localization of dynein reported in earlier studies (Splinter et al., 2012; Kobayashi and Murayama, 2009) may represent an artifact of dynein overexpression (Fig. S3, a and b).

To ascertain that the p150 spots we observed in these cells represented the entire dynactin complex, we used SRRF to visualize p150 in concert with another dynactin subunit, p62 (Fig. 2 c). The p62 subunit of dynactin is located in the pointed end complex of dynactin (Schroer, 2004), and colocalization of p62 with p150 would indicate the presence of the complete dynactin complex. We observed that 49 *±* 12% (mean *±* SD) of the p150 spots colocalized with p62 (*n* = 21,934/44,306 spots from *N* = 2 independent experiments with 59 cells, Fig. 2 d). Additionally, we used SRRF to visualize the localization of p62 on MTs. The presence of p62 on MTs would indicate association with the MT of a subunit which does not normally do so unless it is part of the entire dynactin complex. Therefore, occurrence of p62 on the MT would imply localization of the entire complex on the MT via p150 or dynein. We observed that 74 *±* 18% (mean *±* SD) of the p62 spots (*n* = 59,715/79,639 spots from 1 experiment with 25 cells) were present on MTs (Fig. 2, e and f). To corroborate our results using another super-resolution technique and to ensure our observations were not due to an image processing artifact, we utilized Airyscan confocal microscopy. First, we confirmed specificity of our p150 and p62 antibodies, showing they readily detect p150 and p62 expression constructs (Fig. S3 c). We observed similar results to SRRF using Airyscan confocal microscopy with the p150 and p62 antibodies (Fig. S3, d and e). Therefore, the complete dynactin complex is likely present along the entire length of the MT lattice, including the plus tip. Further, we observed that depletion of p150 through siRNA-mediated silencing reduced the levels of both p150 and p62 along the MT lattice (Fig. S3, f–k).

### Dynactin remains persistently associated with MTs

**Figure 3.**
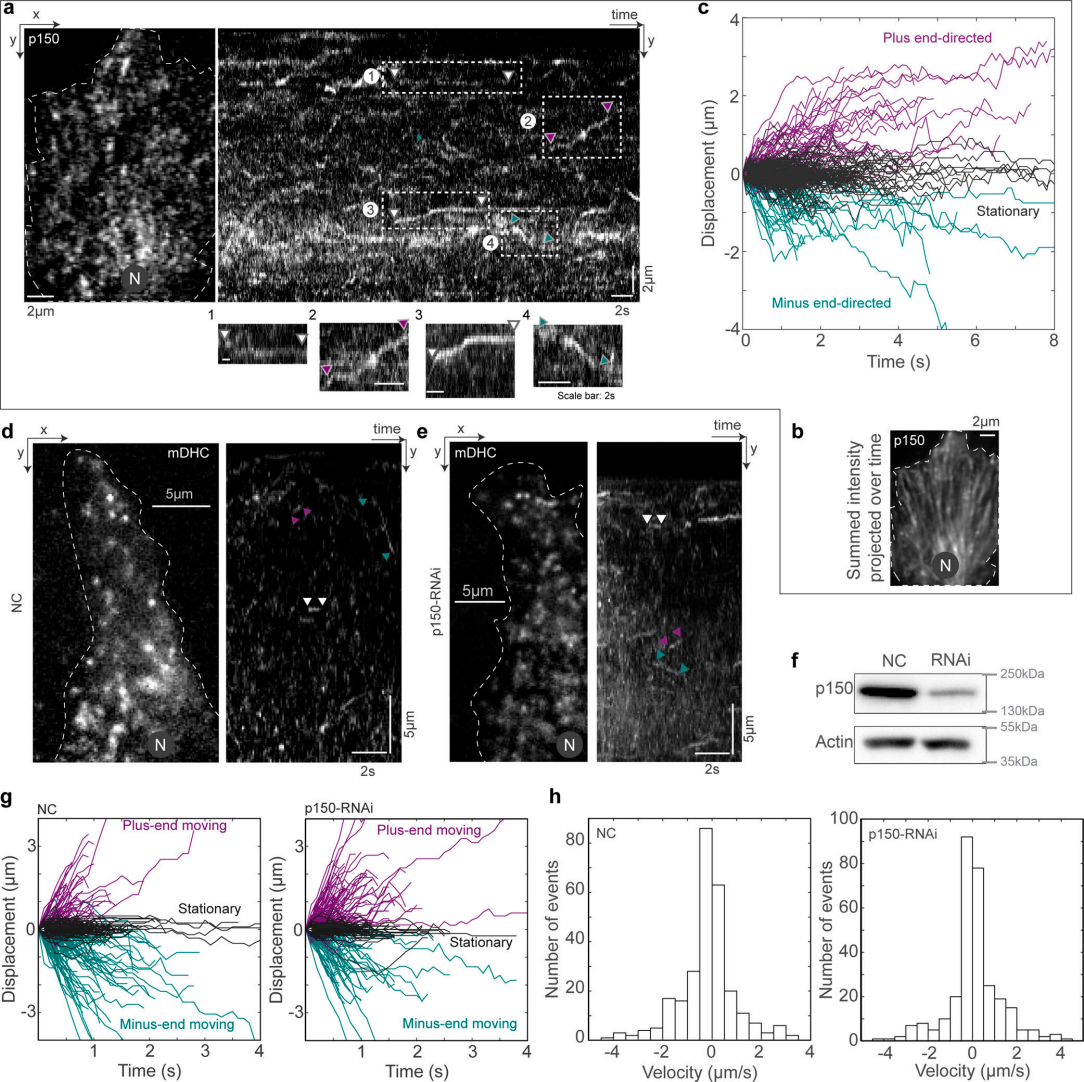
**Dynactin depletion reduced the number of minus end–directed dynein molecules on MT. (a)** HILO image (left) and kymograph (right) of a cell expressing GFP-p150. Representative stationary (switching to stationary from minus [1] or plus end–directed [3]), minus end–directed (4), and plus end–directed events (2) are indicated with the white, teal, and magenta arrowheads, respectively, in the kymograph and in the insets below. **(b)** Summed intensity projection over time (∼40 s) of the GFP-p150 time-lapse in a, showing structures that resemble MTs, indicating the likely persistent association of p150 with MTs. **(c)** Plot of position versus time for the p150 molecules tracked, showing stationary events (gray), minus end–directed events (teal), and plus end–directed events (magenta). *n* = 213 spots from 35 cells across three independent experiments. **(d)** HILO microscopy image (left) from a 10-s-long time-lapse video of mDHC-GFP in cells treated with 25 nM negative control (NC) siRNA (left) and the corresponding kymograph (right). Representative stationary, minus end–directed, and plus end–directed events are indicated with white, teal, and magenta arrowheads, respectively, in the kymograph. **(e)** HILO microscopy image (left) from a 10-s-long time-lapse video of mDHC-GFP in cells treated with 25 nM siRNA against endogenous p150 and the corresponding kymograph (right). Representative stationary, minus end–directed, and plus end–directed events are indicated with white, teal, and magenta arrowheads, respectively, in the kymograph. **(f)** Representative western blot to verify the knockdown of p150 by RNAi. Quantification of western blot confirmed that the RNAi successfully knocked down levels of p150 by an average of 68% (*n* = 2 independent experiments). **(g)** Comparison of displacement versus time plots of single dynein molecules in cells treated with 25 nM NC siRNA (top) and 25 nM siRNA against p150 (bottom). Data for NC obtained from *n* = 274 binding events tracked across ∼60 cells from two independent experiments. Data for RNAi obtained from *n* = 299 binding events tracked ∼60 cells from two independent experiments. Fisher’s exact test yielded a significant difference in dynein behavior in NC and p150-RNAi (P < 0.05). **(h)** Histogram of velocities of mDHC-GFP in the background of NC RNAi (top) and p150 RNAi (bottom; for data from d). In a, b, d, and e, “N” marks the location/direction of the nucleus. Source data are available for this figure: SourceData F3.

We then used HILO microscopy to probe the dynamics of p150 in live cells by imaging fluorescently tagged p150. We observed that p150 spots had residence times on the MT that far exceeded that of dynein, with p150 remaining bound to the MT for 2.5 ± 1.2 s (mean ± SD, *n* = 213 p150 spots from *N* = 3 independent experiments with 35 cells; Fig. 3, a–c and Video 2). This residence time is likely a large underestimate since the background from other p150 spots stably bound to MTs precluded reliable tracking for the entire duration that individual punctae appeared to be on the MT. In any case, this residence time of p150 on the MT is approximately five times that of mDHC-GFP, indicating different dynamics (longer residence time) of dynactin on the MT compared with dynein. We also confirmed that p150’s association with MTs had negligible dependence on dynein by visualizing p150 in cells depleted of hDHC using RNAi (Fig. S3, l–n).

**Video 2.** **Dynamics of p150.** HeLa cell expressing GFP-p150 imaged using HILO microscopy. Unlike dynein, whose association with the MT is short lived, p150 punctae are visible for longer durations. The green arrowhead points to a p150 puncta moving toward the minus end of MT, presumably as part of the tripartite complex. The magenta arrowhead points to a p150 puncta moving away from the nucleus, likely at the MT plus end, and a stationary p150 spot is indicated by the white arrowhead. “N” marks the position/direction of the nucleus. Imaged at 50 fps, movie playback 50 fps. Scale bar: 5 µm. Movie related to Fig. 3, f–h.

Finally, we probed whether the single molecules of dynein that bound to MT and moved to the minus ends (Fig. 1, d and e) were indeed activated, likely upon forming the tripartite complex—if they were, then perturbation of dynactin localization along the MT lattice would result in reduced dynein activity and thus movement toward the minus end. To test this, we depleted p150 using siRNA-mediated silencing and observed single-molecule dynein behavior (Fig. 3, d–h). We indeed observed a reduction in the proportion of dynein molecules that moved toward the MT minus ends with a concomitant increase in the proportion that moved toward the plus ends following p150 depletion (Fig. 3 g). In control cells, 48% of the molecules remained stationary, 34% moved toward the minus ends of the MTs and 18% moved toward the plus ends of the MTs, whereas in cells depleted of siRNA, 50% of the molecules remained stationary, 21% moved toward the minus ends of the MTs and 29% moved toward the plus ends of the MTs. The velocities of the plus end– and minus end–directed runs of dynein confirmed this increase in the number of molecules that moved toward the plus end in p150-depleted cells (Fig. 3 h). Taken together, our results indicate that compared to dynein, the dynactin complex is bound more persistently to the MTs, and that this MT-bound dynactin pool influences the behavior of single dynein molecules binding the MTs.

### Dextran and EGF vesicles associate with cargo adaptors Hook1 and Hook3

**Figure S4.**
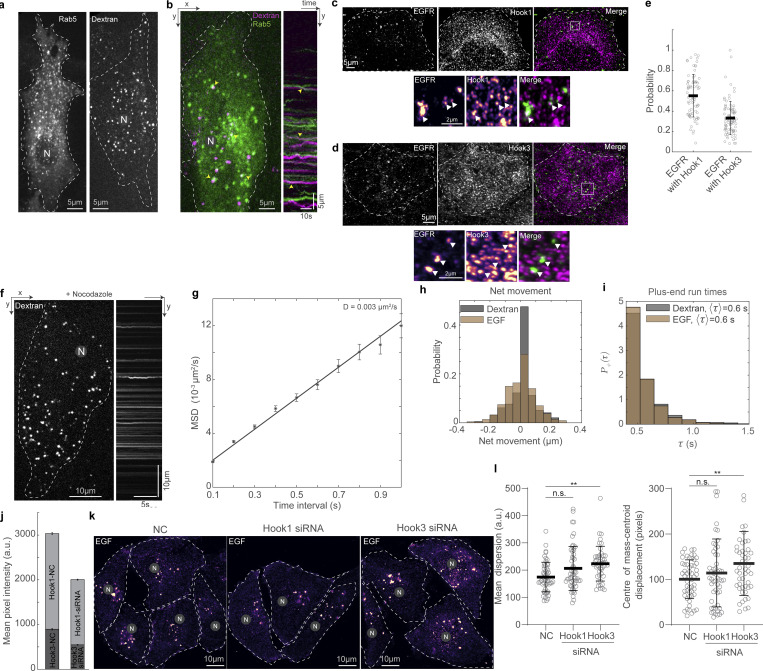
**Dextran as endosomal cargo marker and EGFR’s interaction with Hook proteins. (a)** Spinning disk microscopy images of mCherry-Rab5 (left) and dextran-A647 (right). The signal-to-noise ratio was higher in cells with endocytosed dextran, allowing us to track dextran vesicles with high spatio-temporal resolution and perform dual channel imaging along with single molecules of dynein. **(b)** Spinning disk microscopy image from a 60-s-long time-lapse video of mCherry-Rab5 (green) and dextran-A647 (magenta) in cells (left) and the corresponding kymograph (right). Yellow arrowheads point to colocalized Rab5 and dextran, indicating dextran vesicles were a proxy for early endosomal compartments. In images acquired within 60 min after a 10-min pulse of dextran, 63 *±* 14% of the dextran vesicles were associated with a Rab5 punctae (*n* = 1 25 dextran vesicles from *n* = 1 independent experiment with 17 cells). **(c)** Immunofluorescence images of EGFR (left, green), Hook1 (middle, magenta), and their merge (right) obtained using Airyscan confocal microscopy. The inset (marked with a white box) is depicted at the bottom of the images. EGFR and Hook3 channel insets are depicted as intensity maps and the white arrowheads point to EGFR punctae, some of which colocalize with Hook1. **(d)** Immunofluorescence images of EGFR (left, green), Hook3 (middle, magenta), and their merge (right) obtained using Airyscan confocal microscopy. The inset (marked with a white box) is depicted at the bottom of the images. EGFR and Hook3 channel insets are depicted as intensity maps and the white arrowheads point to EGFR punctae, some of which colocalize with Hook3. **(e)** Plot of the probability of co-occurrence of EGFR with Hook1 and Hook3, showing a slightly higher probability of Hook1 being found on EGFR vesicles compared with Hook3. We confirmed that the colocalization of EGFR with Hook1 and Hook3 was not coincidental by calculating the probability of co-occurrence after flipping the EGFR channel horizontally and proceeding with our analysis. For both Hook1 and Hook3, the colocalization probability with EGFR reduced significantly with the flipped image (flipped EGFR with Hook1: 0.3 *±* 0.2 [mean ± SD]; with Hook3: 0.2 *±* 0.1 [mean ± SD]; both P < 10^−4^ two-sample Kolmogorov–Smirnov test), indicating that the colocalization probability calculated from the original image is a true representation. *n* = ∼25 cells across three independent experiments. **(f)** Spinning disk microscopy image from a 10-s-long time-lapse video of dextran-A647 in cells treated with 10 µM nocodazole (left) and the corresponding kymograph (right). The kymograph shows abrogation of directed transport, as expected, upon MT depolymerization. **(g)** Mean squared displacement (MSD) analysis of dextran vesicles tracked in cells treated with 10 µM nocodazole for >30 min. The MSD data of dextran vesicles was fit to *<x2> = 4Dt + c*, and the intercept *c* was estimated to 0.0008. The diffusion coefficient *D* was 0.003 µm^2^/s, indicating that even in the absence of MTs, intracellular crowding likely prevented the dextran vesicles from diffusing away. (*n* = 804 dextran vesicles from *n* = 1 independent experiment with 24 cells.) Error bars represent SEM. **(h)** Histogram of net movement of dextran (gray) and EGF (brown) endosomes, indicating that EGF-containing endosomes undertook more net minus end–directed movements in these 3-min time-lapse videos. **(i)** Probability distribution (*Ρ*_*+*_*(τ)*) of the plus end–directed runs for dextran (gray) and EGF (brown) vesicles. The plus end run time for both dextran and EGF were calculated to be 0.6 *±* 0.2 s. **(j)** Quantification of the mean pixel intensity of Hook1 (light gray) and Hook3 (dark gray) in NC and Hook1/Hook3 siRNA cells. siRNA of Hook1 resulted in a reduction of Hook1 by 33.9% and reduction of Hook3 by 37.9% (*n* > 100 cells across *n* = 2 independent experiments). **(k)** Representative images of EGF in control cells (left, “NC”), and cells with Hook1 siRNA (middle) and Hook3 siRNA (right), fixed 20 min after the addition of fluorescent EGF. Dashed lines indicate cell boundaries. **(l)** Plots of mean dispersion of EGF vesicles (left) and displacement between the center of mass of EGF vesicles and the cell centroid (right) in NC, Hook1 siRNA, and Hook3 siRNA cells. “n.s.” represents no significant difference and ** represents P < 0.01 (*n* > 45 cells across *n* = 3 independent experiments, Kruskal–Wallis test). In a, b, f, and k, “N” marks the location/direction of the nucleus. Error bars in e, j, and l represent SD.

We next sought to understand how dynein interacted with the third component of the active complex—the cargo adaptors. The Hook family of endosomal cargo adaptors have been observed to remain persistently bound to their respective cargo in fungi (Bielska et al., 2014) and in mammalian cells (Olenick et al., 2019; Christensen et al., 2021). Therefore, we used endosomal cargo as a proxy for the cargo adaptor, which allowed us to visualize the movement of the entire cargo and adaptor complex. To avoid artifacts from overexpressing fluorescently tagged Rab5 to visualize early endosomes (Nielsen et al., 1999), we employed cells that had taken up Alexa^647^-conjugated 10 kD dextran or epidermal growth factor (EGF) (Fig. S4 a).

We first confirmed that these cargoes transited to early endosomes in our experimental time frame (5–20 min following fluorescent cargo uptake). Following this short pulse and chase, we observed dextran in Rab5-positive compartments (Fig. S4 b and Video 3). EGF has also been shown to be in Rab5-positive compartments within the timescales of our chase observations (Leonard et al., 2008). Next, we queried if the known endosomal cargo adaptors Hook1 and Hook3 were associated with these endosomes. We confirmed that both Hook1 and Hook3 are associated with the EGF receptor (EGFR) cargo used in our study by visualizing the colocalization of the adaptors with EGFR endosomes in immunofluorescence (Fig. S4, c–e). We quantified that EGFR-containing endosomes associated with Hook1 with a probability of 55 ± 21% (*n* = 75 cells from *N* = 3 independent experiments) and with Hook3 with a probability of 33 ± 16% (*n* = 90 cells from *N* = 3 independent experiments, Fig. S4 e) 20 min following addition of 1 nM EGF. This indicates a high degree of co-occurrence of Hook1/Hook3 with EGFR. Recent studies have also demonstrated that depletion of Hook1, 2, and 3 reduces EGF colocalization with lysosomes (Xu et al., 2008). Together, our immunofluorescence data show that both dextran- and EGF-containing endosomes are of early endosomal identity and co-occur with Hook1 and Hook3 in our experimental time frame. With previous studies in mammalian cells indicating that Rab5-positive endosomes are likely to be persistently associated with cargo adaptors Hook1 and Hook3 (Christensen et al., 2021), our results therefore suggest that the dextran- and EGF-containing vesicles are useful proxies to study the movement of the cargo–adaptor complex.

**Video 3.** **Dextran is in Rab5**^**+**^ **compartments.** HeLa cell expressing mCherry-Rab5 (green) and after taking up A647 tagged 10 kD dextran (magenta) imaged using spinning disk microscopy. The white arrowheads point to dextran and Rab5 that colocalized for the duration of the video, indicating that dextran could be used as a marker for early endosomes. Imaged at 1 fps, movie playback 6 fps. Scale bar: 5 µm. Movie related to Fig. S4 b.

### Endosomes remain close to MTs and move in short bursts

**Figure 4.**
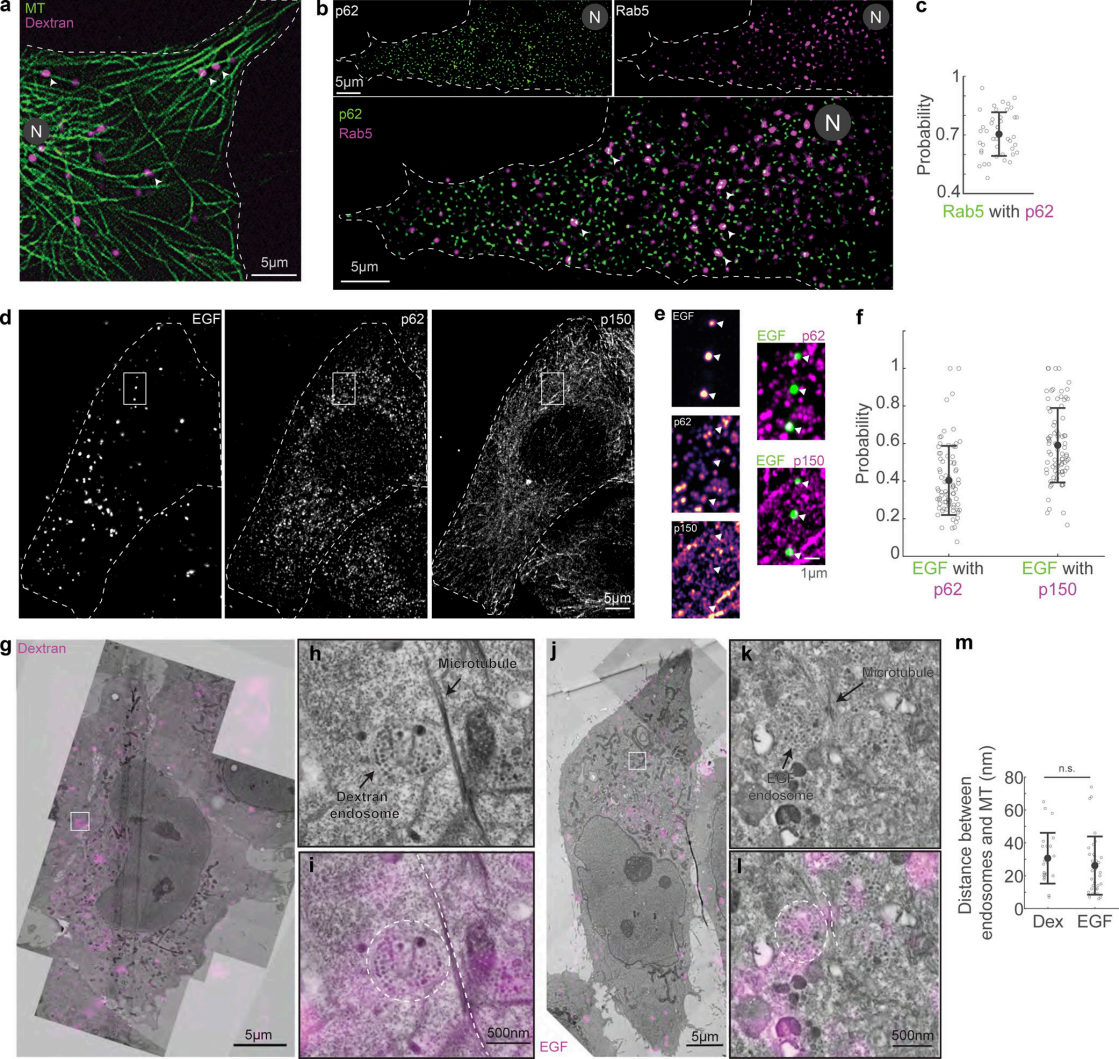
**Endosomes remain close to MTs. (a)** Spinning disk + SRRF image of MTs (green) and dextran vesicles (magenta) in live cells. The white arrowheads indicate representative vesicles on the MT. **(b)** Spinning disk + SRRF image of p62 (top left), Rab5 vesicles (top right) in live cells, and their merge (bottom, p62 in green and Rab5 in magenta). The white arrowheads in the merged image indicate representative vesicles that colocalize with p62 and are shown as green and magenta arrowheads in the p62 and Rab5 images, respectively. In a and b, “N” marks the location/direction of the nucleus. **(c)** Plot of the probability of co-occurrence of p62 with Rab5. *n* = ∼40 cells across two independent repeats. **(d)** Fluorescence images of EGF (left), p62 (middle), and p150 merge (right) obtained using Airyscan confocal microscopy. **(e)** The insets marked with a white box in d are depicted; EGF, p62, and p150 channel insets are depicted as intensity maps and the white arrowheads point to EGF punctae, some of which colocalize with p62 and p150. **(f)** Plot of the probability of co-occurrence of EGF with p62 and p150, indicating a high likelihood of dynactin being present in a complex with endosomal cargo. *n* = ∼60 cells across three independent repeats. **(g)** Overlay of confocal images of MT (green) and dextran vesicles (magenta), and EM images of the same cell (gray). **(h)** EM image of the region indicated with the white square in g. **(i)** Merge of h and confocal fluorescence image of dextran (magenta) of the region in h showing a representative MT (dashed white line) and dextran endosome (dashed white circle). **(j)** Overlay of confocal images of MT (green) and EGF vesicles (magenta), and EM images of the same cell (gray). **(k)** EM image of the region indicated with the white square in j. **(l)** Merge of k and confocal fluorescence image of EGF (magenta) of the region in k showing a representative MT (dashed white line) and EGF endosome (dashed white circle). **(m)** Quantification of the measured distance between dextran (“Dex”) and EGF endosomes and MTs. “n.s.” indicates no significant difference (P = 0.3), one-way ANOVA, Tukey Kramer post-hoc test. *n* = 6 cells from two independent repeats. Error bars represent SD.

We then probed the localization of dextran and EGF vesicles with respect to the MT. First, we imaged dextran vesicles and MTs in live cells and observed that the vesicles were in proximity to the MTs (Fig. 4 a). We also observed that the dextran vesicles remained close to MTs even while they had no observable tether to the MTs via motor proteins and were therefore stationary (Video 4) in our minute-long live-cell time-lapse images. We then used SRRF to visualize Rab5 in concert with p62 and observed that ∼70% of the Rab5 spots colocalized with p62 (*n* = 8,795/12,848 vesicles from *N* = 2 independent experiments with >20 cells each), indicating that cargo and dynactin are in proximity to each other on the MT (Fig. 4, b and c). We found that dextran is not retained well after methanol fixation, and hence we were unable to visualize dextran with p150 or p62 using immunofluorescence. In its place, we visualized EGF vesicles and p62/p150 using Airyscan microscopy (Fig. 4, d and e) and observed that ∼40% (*n* = 15,156/46,571 vesicles) of EGF vesicles localized with p62, whereas ∼60% colocalized with p150 (*n* = 25,015/46,571 vesicles, from *N* = 3 independent experiments with >20 cells each, Fig. 4 f). Finally, we employed CLEM to visualize the location of dextran and EGF endosomes with respect to MTs. We observed that in both instances, the endosomes were along MTs, indicating that endosomal cargo remained within ∼30 nm of MTs (dextran: 31 ± 15 nm, from 23 endosomes, *N* = 5 cells; EGF: 26 ± 18 nm, from 33 endosomes, *N* = 6 cells), likely along with dynactin (Fig. 4, g–m). Interestingly, the size of the dynactin complex is ∼35 nm (Hodgkinson et al., 2005), comparable to the distances observed between dextran and EGF endosomes from MTs, indicating dynactin may hold endosomes to MTs.

**Video 4.** **Cargo–MT interaction.** HeLa cell expressing mCherry-tubulin (green), after uptake of A647-tagged 10 kD dextran (magenta) imaged using spinning disk microscopy. The white arrowheads point to dextran vesicles that remained close to the MTs even when stationary. Imaged at 1 fps, movie playback 3 fps. Scale bar: 5 µm. Movie related to Fig. 4 a.

To understand if endosomal vesicles could diffuse away from their original locations on the MT upon motor unbinding, we depolymerized MTs using nocodazole and visualized the subsequent movement of the vesicles (Fig. S4 f and Video 5). Confirming previous findings on Rab5-positive compartments (Flores-Rodriguez et al., 2011; Zajac et al., 2013), we measured a lower diffusion coefficient for the vesicles in the absence of MTs (Fig. S4 g), likely implicating the role of high intracellular crowding in constraining vesicle diffusion, also previously noted by others (Zajac et al., 2013; Weiss et al., 2004; Ernst et al., 2012; Szymanski and Weiss, 2009; Sokolov, 2012). This intracellular crowding may also obviate the need for dynactin to act as a tether between endosomes and MTs.

**Video 5.** **Intracellular crowding prevents cargo diffusion.** HeLa cell after uptake of A647-tagged 10 kD dextran treated with 10 µM nocodazole to depolymerize the MTs. As expected, directed movement of the dextran vesicles was completely abrogated. The diffusion of the vesicles was also insignificant, indicating that intracellular crowding likely prevented cargo diffusion away from the MTs. “N” marks the position/direction of the nucleus. Imaged at 50 fps, movie playback 50 fps. Scale bar: 5 µm. Movie related to Fig. S4 f.

**Figure 5.**
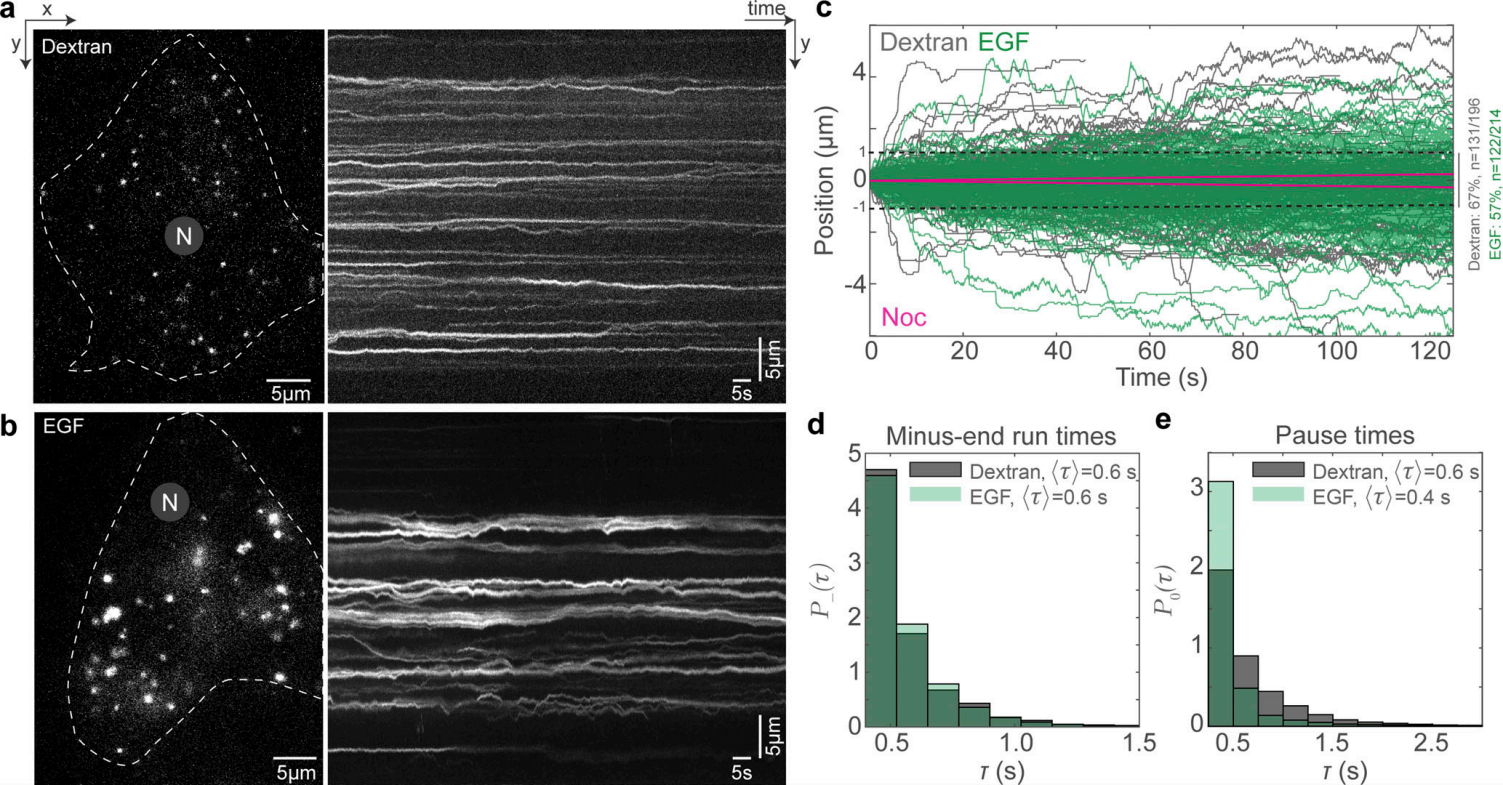
**Dextran and EGF vesicles display different pause times. (a)** HILO image from a time-lapse of dextran vesicles in HeLa cells (left) and the corresponding kymograph (right). **(b)** HILO image from a time-lapse of EGF vesicles in HeLa cells (left) and the corresponding kymograph (right). **(c)** Plot of position versus time of dextran vesicles (gray) and EGF (green) in HeLa cells, and the predicted movement of endosomes after nocodazole treatment in the absence of MTs (magenta). The dashed black lines indicate absolute movement of 1 µm, with 67% of dextran vesicles and 57% of EGF vesicles moving <1 µm during the time-lapse imaging. **(d)** Probability distribution *P−*(*τ*) of the minus end–directed run times for dextran (gray) and EGF (green) vesicles. *n* = 196 tracks from two independent experiments. **(e)** Probability distribution P0(*τ*) of the pause times for dextran (gray) and EGF (green) vesicles. In a and b, “N” marks the location/direction of the nucleus. In d and e, the average run/pause time (〈τ〉) for dextran and EGF vesicles are indicated. *n* = 214 tracks from two independent experiments.

Small molecular weight dextran enters the cell through all endocytic mechanisms via fluid-phase endocytosis, and uptake is therefore relatively independent of receptor-mediated endocytosis (Rennick et al., 2021; Li et al., 2015). On the other hand, EGF is a ligand that binds to the EGFR on the cell membrane, which at the concentrations used in this study is taken up by clathrin-mediated endocytosis (Goh et al., 2010). Both these cargoes end up at lysosomes near the minus ends of MTs over different timescales: dextran requires hours (Humphries et al., 2011) while EGF requires tens of minutes to reach the lysosome (Futter et al., 1996). We sought to understand how dynein’s short run times act to ensure cargoes reach the lysosomes in different timescales. We visualized dextran and EGF vesicles in 100-s time-lapses and observed that the directed runs were sparse (Fig. 5, a and b; and Videos 6 and 7), with only ∼33% of dextran vesicles (65/196) and ∼43% EGF vesicles (92/214) moving >1 µm during this time (Fig. 5 c). Strikingly, both the dextran and EGF vesicles displayed uninterrupted minus end–directed runs that lasted only *∼*0.6 s on average (Fig. 5 d; 0.6 *±* 0.2 s [mean *±* SD], *n* = 196 and 214 for dextran and EGF vesicles, respectively, from *N* = 2 independent experiments). Interestingly, the time between two consecutive runs, the pause time, reflected the timescales of minus end–directed movement of EGF and dextran, with EGF vesicles having an average pause time that was 30% shorter than that of dextran vesicles (0.4 versus 0.6 s, Fig. 4 e). The net distance thus moved toward the minus end was higher for EGF vesicles than dextran vesicles, whereas the plus end run times for both were similar (Fig. S4, h and i). These results are comparable with the run-and-pause behavior observed for endosomal cargo in previous studies (Flores-Rodriguez et al., 2011; Zajac et al., 2013). By visualizing EGFR in cells subjected to RNAi of Hook1 and Hook3 and fixed 20 min following 1 nM EGF addition, we confirmed earlier findings (Xu et al., 2008) that at least Hook3 is involved in the movement of these compartments, with EGFR endosomes in Hook3 siRNA cells being significantly dispersed from the cell center compared to control siRNA treated cells (Fig. S4, j–l). These results indicate our observations of endosomal movement represent genuine activating adaptor-mediated motor-driven movement events.

**Video 6.** **Movement of dextran vesicles in HeLa cells.** Cell after uptake of A647-tagged 10 kD dextran imaged using HILO microscopy. Imaged at 8 fps, movie playback 100 fps. Scale bar: 5 µm. Movie related to Fig. 5 a.

**Video 7.** **Movement of EGF vesicles in HeLa cells.** Cell after uptake of A647-tagged EGF imaged using HILO microscopy. Imaged at 8 fps, movie playback 100 fps. Scale bar: 5 µm. Movie related to Fig. 5 b.

### Transient dynein attachment to cargo complexes is sufficient to drive typical cargo trafficking

**Figure S5.**
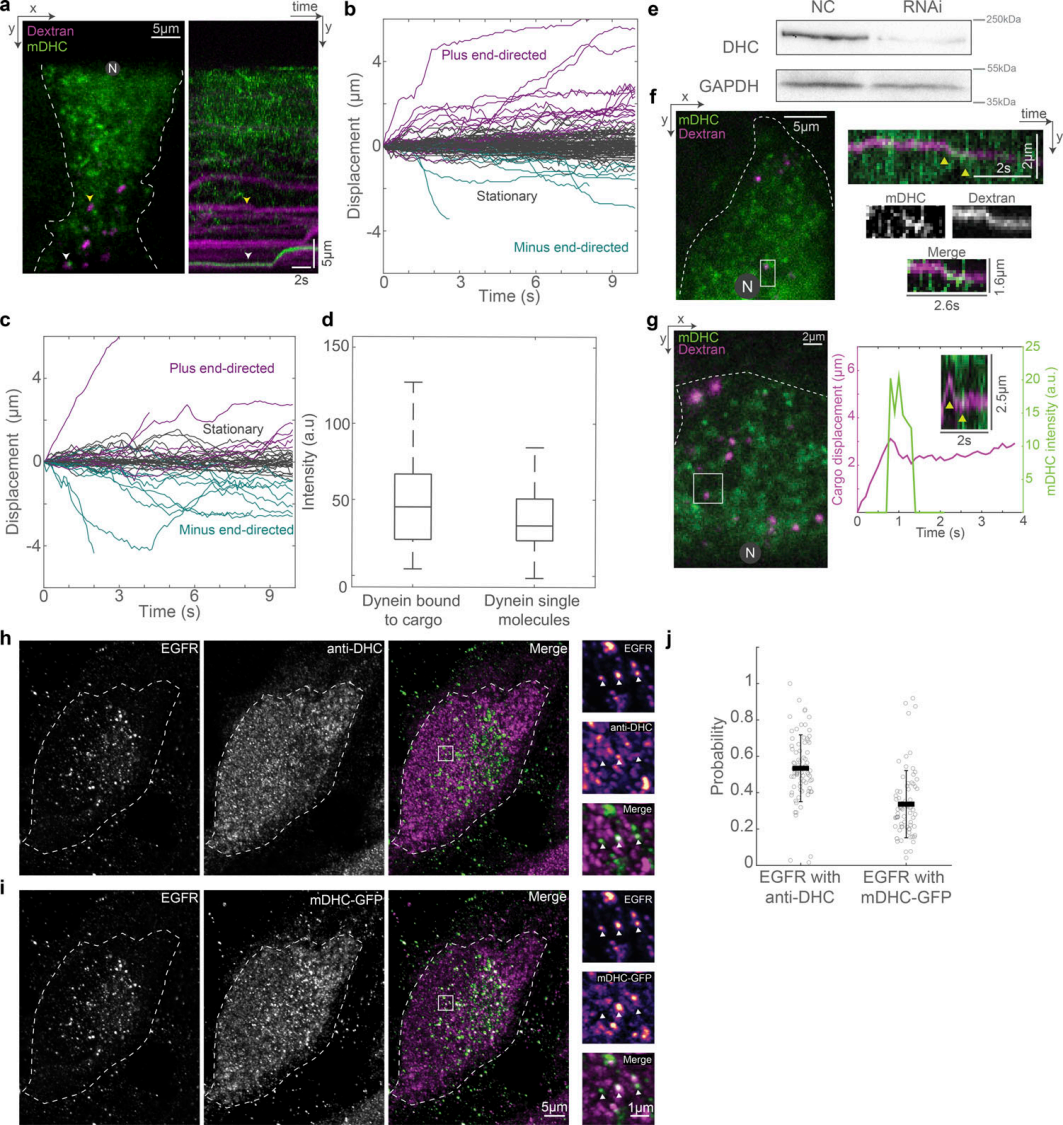
**Dextran and EGFR’s interaction with dynein. (a)** HILO microscopy image (left) from a 10-s-long time-lapse video of mDHC-GFP (green) and dextran-A647 (magenta) and the corresponding kymograph (right). The yellow arrowhead points to a dextran vesicle with no dynein fluorescence and the white arrowhead points to a dextran vesicle with associated dynein intensity. The kymograph shows that a dextran vesicle with dynein bound to it (white arrowhead) made a short run toward the MT minus end. The dextran vesicle without visible dynein intensity (yellow arrowhead) remained stationary. **(b)** Displacement versus time plot of dextran vesicles without associated dynein intensity. **(c)** Displacement versus time plot of dextran vesicles with visible dynein intensity. Comparing b and c, while 28% of dextran vesicles with visible dynein intensity moved toward the MT minus ends (*n* = 12/44 vesicles), only 10% of those without associated dynein intensity moved to the minus end of MTs (*n* = 11/114 dextran vesicles; *n* = 2 independent experiments with >25 cells). In other words, of all the minus end–directed dextran vesicles observed in these experiments (all the teal-colored traces in b and c), ∼52% (*n* = 12/23 vesicles) had associated dynein intensity. Fisher’s exact test yielded a significant difference in dynein-associated and non-dynein-associated endosome behavior (P < 0.005). **(d)** Box plots comparing the intensity of dynein on dextran vesicles and intensity of single molecules of dynein (measured from binding events) showed that there were one to two dynein molecules per dextran vesicle (*n* = 44 dextran vesicles with dynein intensity, *n* = 115 single molecules of dynein from *n* = 2 independent experiments with >25 cells). **(e)** Analysis of b–d was done in cells expressing mDHC-GFP and treated with 25 nM siRNA against endogenous hDHC. Quantification of western blot confirmed that the RNAi successfully knocked down levels of DHC by 85%. **(f)** HILO microscopy image (left) from a time-lapse video of mDHC-GFP (green) and dextran-A647 (magenta), and the kymograph corresponding to the region marked with the white square (right). The yellow arrowheads point to a short run of the dextran vesicle toward the MT minus end upon binding of a dynein molecule; the portion of the kymograph with the run is enlarged and shown on the bottom right. A longer run in the same cell has been depicted in Fig. 6 a. **(g)** HILO microscopy image (left) from a time-lapse video of mDHC-GFP (green) and dextran-A647 (magenta) in cells with RNAi-mediated depletion of hDHC, and the kymograph and plots corresponding to the region marked with the white square (right). The yellow arrowheads point to a short run of a previously plus end–directed dextran vesicle toward the MT minus end upon binding of a dynein molecule. **(h)** Immunofluorescence images of EGFR (left, green), DHC (middle, magenta), and their merge (right) were obtained using Airyscan confocal microscopy. The inset (marked with a white box) is depicted to the right of the images. EGFR and anti-DHC channel insets are depicted as intensity maps and the white arrowheads point to EGFR punctae, some of which colocalize with anti-DHC. **(i)** Fluorescence images of anti-EGFR (left, green), mDHC-GFP (middle, magenta), and their merge (right) obtained using Airyscan confocal microscopy. The inset (marked with a white box) is depicted to the right of the images. EGFR and mDHC-GFP channel insets are depicted as intensity maps and the white arrowheads point to EGFR punctae, some of which colocalize with mDHC-GFP. **(j)** Plot of the probability of co-occurrence of EGFR with anti-DHC and mDHC-GFP 53 ± 18% of EGFR containing endosomes colocalized with DHC (human/mouse), and 33 ± 18% EGFR vesicles colocalized with mDHC-GFP (*n* = 76 cells across *n* = 3 independent experiments). This indicates that we are likely missing ∼40% ([53-33]/55) of the runs by these cargo (and presumably other cargo moved by dynein) in our imaging, which could explain the fewer runs we see in our imaging. Error bars represent SD. In a, f, and g, “N” marks the location/direction of the nucleus. Source data are available for this figure: SourceData FS5.

**Figure 6.**
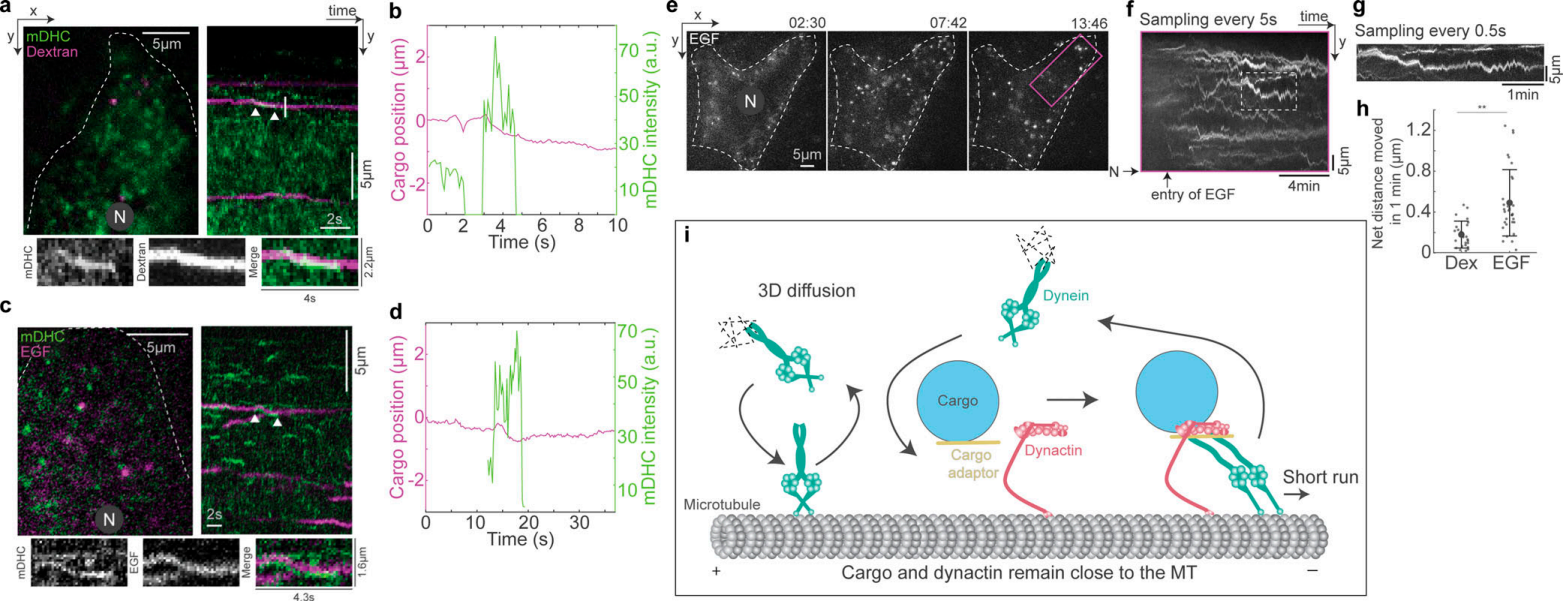
**Cargo capture and dynein activation are coupled. (a)** Image from the first frame of a time-lapse video (left) of dynein (green) and dextran (magenta), and the corresponding kymograph (right). The white arrowheads point to dynein and dextran vesicles moving together toward the minus end and are shown separately in the images on the bottom. See also Fig. S5 f for an example of another such event in the same cell. **(b)** Plot of position versus time of the dextran vesicle (magenta) indicated in a, alongside the intensity of dynein on that vesicle (green), showing a short minus end–directed run of the vesicle upon dynein binding. *n* = ∼15 cells across two independent repeats. **(c)** Image from the first frame of a time-lapse video (left) of dynein (green) and EGF (magenta), and the corresponding kymograph (right). The white arrowheads point to dynein and EGF vesicle moving together toward the minus end and are shown separately in the images on the bottom. **(d)** Plot of position versus time of the EGF vesicle (magenta) indicated in c, alongside the intensity of dynein on that vesicle (green), showing a short minus end–directed run of the vesicle upon dynein-binding. *n* = ∼15 cells across two independent repeats. **(e)** Images from time-lapse video of EGF entry and subsequent movement within cells. Time is indicated above the images in min:s. **(f)** Kymograph of the region indicated by the magenta rectangle in e, with the time-lapse images sampled every 5 s showing entry of EGF and the net movement of endosomes toward the nucleus. **(g)** Kymograph of the trajectory marked by the white dashed rectangle in f, where the time-lapse images were sampled every 0.5 s. **(h)** Quantification of the net distance moved by dextran (“Dex”) and EGF endosomes in 1 min. Asterisks indicate significant difference, P = 4 × 10^−5^, one-way ANOVA, Tukey Kramer post-hoc test. *n* = 24 dextran endosomes from 12 cells across two independent repeats and 32 EGF endosomes from nine cells across two independent repeats. Error bars represent SD. **(i)** Schematic of dynein’s cargo search mechanism: stochastic binding of dynein to the MT at a location proximal to the cargo–adaptor complex leads to a short minus end–directed run, which terminates upon the unbinding of dynein. In a, c, and e, “N” marks the location/direction of the nucleus.

Thus far, we have discovered that dynein transiently interacts with the MTs while dynactin–cargo complexes are maintained close to MTs. We therefore sought to understand how dynein interacted with the dynactin–cargo complexes on MTs. First, we performed dual-color imaging of dynein and endosomal vesicles and observed that vesicles that had dynein signal were, as expected, more likely to move toward the minus end of MTs (Fig. S5, a–c). Next, by comparing the intensity of mDHC-GFP on endosomal vesicles to single molecule binding events in cells that were depleted of hDHC (Fig. S5, d and e), we estimated that on average there were 1–2 mDHC-GFP molecules bound to a vesicle (*n* = 44 endosomes, from *N* = 2 independent experiments with >25 cells). Finally, to verify if single molecules of dynein could be activated and perform minus end–directed movement when they stochastically bound to MTs and encountered cargo complexes, we performed fast dual-color HILO imaging of single dynein molecules, along with dextran and EGF vesicles. In these videos (Videos 8 and 9), we observed instances where previously stationary dextran and EGF vesicles started moving together with mDHC-GFP toward the MT minus ends upon dynein binding (Fig. 6, a–d, *n* = 8/9 and *n* = 3/3 such events for dextran and EGF, respectively, from *N* = 2 independent experiments with at least 15 cells). We note that the examples in Video 8 and 9 are rarer, longer events, and we additionally observed shorter events of dynein binding and effecting dextran and EGF vesicle movements (Fig. S5, f and g, *n* = 19 and *n* = 14 short events for dextran and EGF, respectively, from *N* = 2 independent experiments with at least 15 cells). Additionally, by comparing colocalization of mDHC-GFP and anti-m/hDHC staining (for total dynein) on EGFR vesicles, we estimated that due to the presence of unlabeled endogenous dynein on endosomal cargoes, we were unable to account for ∼40% of dynein driven events in our imaging (Fig. S5, h–j).

**Video 8.** **Activation of dynein upon dextran vesicle binding.** HeLa cell expressing mDHC-GFP (green), after uptake of A647-tagged 10 kD dextran (magenta) imaged using HILO microscopy. The white arrowhead points to a dextran vesicle onto which a dynein molecule bound (indicated by an abrupt appearance of intensity in the GFP channel) followed by movement of the dynein–cargo complex toward the minus end of MTs. “N” marks the position/direction of the nucleus. Imaged at 10 fps, movie playback 10 fps. Scale bar: 5 µm. Movie related to Fig. 6, a and b.

**Video 9.** **Activation of dynein upon EGF vesicle binding.** HeLa cell expressing mDHC-GFP (green), after uptake of A647-tagged EGF (magenta) imaged using HILO microscopy. The white arrowhead points to an EGF vesicle onto which a dynein molecule bound (indicated by an abrupt appearance of intensity in the GFP channel) followed by movement of the dynein–cargo complex towards the minus end of MTs. “N” marks the position/direction of the nucleus. Imaged at 10 fps, movie playback 10 fps. Scale bar: 5 µm. Movie related to Fig. 6, c and d.

We next queried whether the transient movements driven by dynein were sufficient to drive EGF and dextran vesicles to perinuclear regions of the cell in time frames consistent with their delivery to lysosomes. We visualized the uptake and subsequent movement of EGF endosomes over a 17-min period using HILO microscopy. We observed robust movement of the EGF endosomes in the net minus end direction during the duration of imaging (Fig. 6, e and f; and Video 10). Strikingly, movements that appeared as long range at a low sampling rate (Fig. 6 f) showed intrinsic short runs when sampled at a higher rate (Fig. 6 g), confirming the short-range movements such as those observed in Fig. 5 are cumulatively sufficient to drive expected cargo transport.

**Video 10.** **EGF endosomes undertake large movements toward the nucleus.** HeLa cell after uptake of A647-tagged EGF (magenta) imaged using HILO microscopy over an ∼17-min time period. “N” marks the position/direction of the nucleus. Imaged at 2 fps, movie playback 100 fps. Scale bar: 5 µm. Movie related to Fig. 6, e–h.

**Figure S6.**
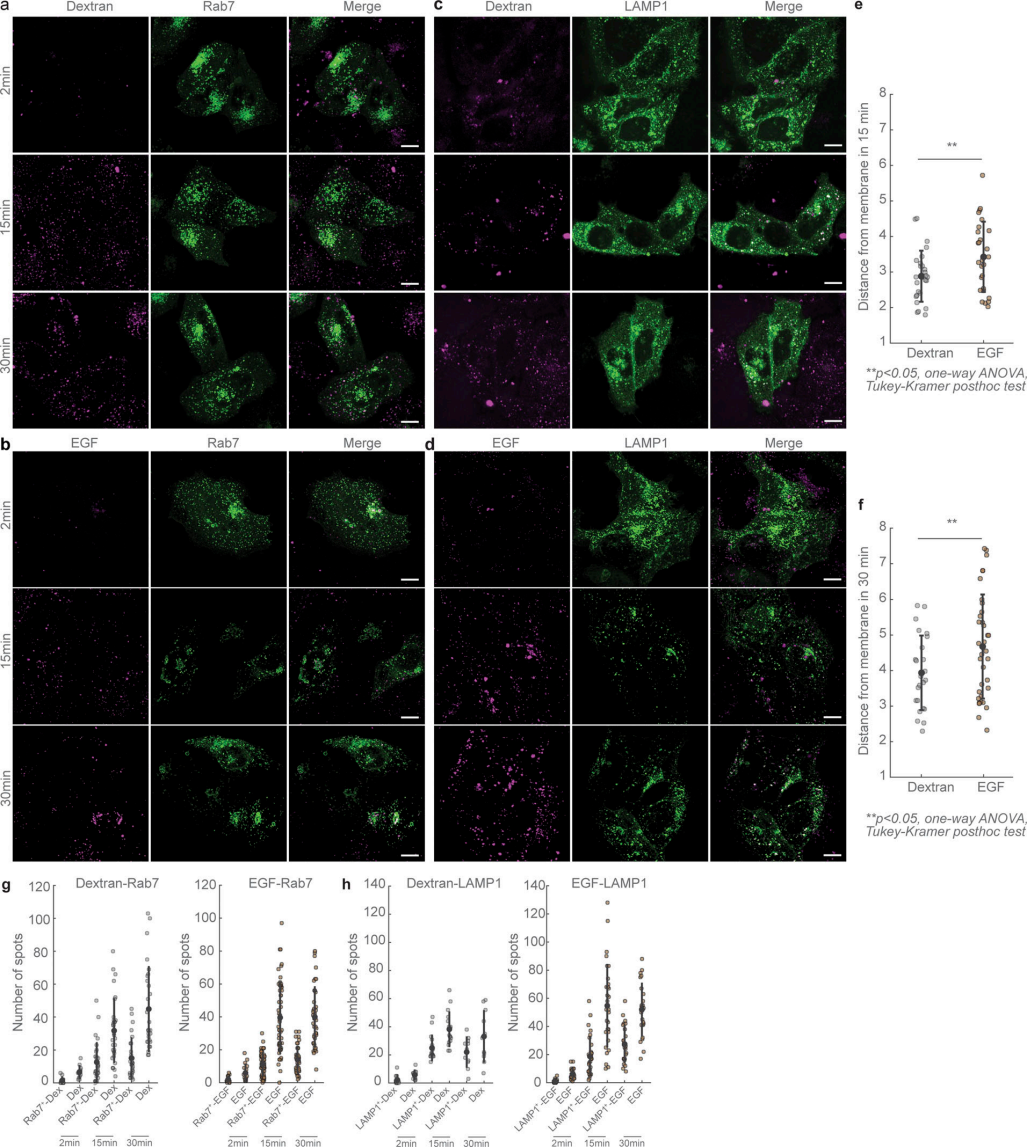
**Net movement of dextran and EGF endosomes over time. (a)** Airyscan confocal images of dextran-AF647 (magenta, left), Rab7-GFP (green, center), and their merge in HeLa cells fixed 2 min (top), 15 min (middle), and 30 min (bottom) after introduction of dextran to the cells. **(b)** Airyscan confocal images of EGF-AF647 (magenta, left), Rab7-GFP (green, center), and their merge in HeLa cells fixed 2 min (top), 15 min (middle), and 30 min (bottom) after introduction of EGF to the cells. **(c)** Airyscan confocal images of dextran-AF647 (magenta, left), LAMP1-GFP (green, center), and their merge in HeLa cells fixed 2 min (top), 15 min (middle), and 30 min (bottom) after introduction of dextran to the cells. **(d)** Airyscan confocal images of EGF-AF647 (magenta, left), LAMP1-GFP (green, center), and their merge in HeLa cells fixed 2 min (top), 15 min (middle), and 30 min (bottom) after introduction of EGF to the cells. **(e)** Quantification (mean *±* SD) of the distance of dextran and EGF vesicles from the membrane in cells fixed 15 min after the introduction of AF647-labeled dextran or EGF. Each dot represents the mean distance of several endosomes from an individual cell (*n* = 29 for dextran data, and *n* = 29 cells for EGF data, from *n* = 3 independent experiments). **(f)** Quantification (mean ± SD) of the distance of dextran and EGF vesicles from the membrane in cells fixed 30 min after the introduction of AF647-labeled dextran or EGF. Each dot represents the mean distance of several endosomes from an individual cell (*n* = 26 cells for dextran data, and *n* = 37 cells for EGF data, from *n* = 3 independent experiments). **(g)** Plot of the number of Rab7^+^ dextran vesicles compared to the number of dextran vesicles (left), and plot of number of Rab7^+^ EGF vesicles compared with the number of EGF vesicles (right) at 2, 15, and 30 min after introduction of AF647-labeled dextran or EGF to HeLa cells. **(h)** Plot of the number of LAMP1^+^ dextran vesicles compared with the number of dextran vesicles (left), and plot of the number of LAMP1^+^ EGF vesicles compared with the number of EGF vesicles (right) at 2, 15, and 30 min after introduction of AF647-labeled dextran or EGF to HeLa cells. Scale bars in a–d: 10 µm; error bars in e–h represent SD.

We measured the net movement of dextran and EGF endosomes in these longer time-lapses and observed that these endosomes moved a net distance of 0.2 *±* 0.1 and 0.5 *±* 0.3 µm (mean *±* SD, from *n* = 24 dextran endosomes from 12 cells, *N* = 2, and *n* = 32 EGF endosomes from 9 cells, *N* = 2) in 1 min, respectively (Fig. 6 h). Considering the average distance between the cell periphery and a lysosome to be 10 µm, a dextran endosome in our experiments would require 50 min and an EGF endosome 20 min on average to reach the lysosome, which are in agreement with previously measured timescales of movement of these cargoes (Futter et al., 1996; Humphries et al., 2011). To confirm that our experimental conditions were optimized for observing these expected trafficking time frames, we performed additional quantification of the distance of dextran- and EGF-containing endosomes from the cell periphery over time (Fig. S6, a–f), as well as their co-occurrence with Rab7-positive and LAMP1-positive compartments, i.e., late endosomes and lysosomes, respectively. These investigations revealed that the behavior of these endosomes was in agreement with previous literature (Futter et al., 1996; Humphries et al., 2011), wherein EGF endosomes occurred at larger distances from the periphery, and also were more likely to be Rab7 and LAMP1 than dextran-containing endosomes at the same time point after internalization (Fig. S6, g and h). Altogether, the higher temporal resolution of our experiments enabled us to discern the dynein-driven stop-and-go behavior of endosomal cargo that is ultimately sufficient to drive movement to its intracellular destination in time frames consistent with those previously observed.

### Conceptual model of dynein’s cargo attachment and subsequent movement within cells

Taken together, we observed that (i) single molecules of dynein bind and unbind stochastically with MTs (Fig. 1 b), (ii) if a dynactin–cargo complex is found close to this attachment location of dynein to the MT, a minus end–directed run of the dynactin–cargo–motor complex is initiated (Fig. 6, a–d), (iii) the detachment of dynein from the cargo concludes this run (Fig. 6 i), (iv) the detached dynein motor is free to diffuse back into the cytoplasm, whereas dynactin and the cargo remain paused and remain close to the MT (Fig. 4), (v) the long-range movement of endosomal cargo likely requires the repeated binding–unbinding of dynein to the dynactin–cargo complex, (vi) the resulting motion of the cargo consists of short minus (and plus) end–directed runs punctuated by long pauses (Fig. 5 c).

Our experimental techniques—HILO, spinning disk microscopy, SRRF, Airyscan, and CLEM—provided high spatiotemporal data for the dynamics of motors and dynactin–cargo complexes. In fact, this not only allowed us to quantify the stochastic kinetics of individual dynein motors to extract its detachment rates (Fig. 1 f) but also allowed us to explore the long-time dynamics of cargo with high precision (Fig. 5 c and Fig. 6, e–g).

## Discussion

In this work, we established a technique to visualize single molecules of dynein in living cells and observed their interaction with the MT and other components of the tripartite complex required for dynein’s activation. We discovered distinct localization and kinetics of dynein, dynactin, and the cargo complex. While dynein interacted stochastically and transiently with the MT, dynactin and cargo remained persistently bound and in proximity to the MT. When dynein interacted with a dynactin–cargo complex upon MT binding, the entire complex moved toward the minus end of MTs in a short run lasting a little over half a second. Repeated rounds of such stochastic interactions of the motor with the MT and cargo complex are likely required for long-range movement of cargoes. Taken together, we demonstrate that stochastic interactions of motors with MTs and cargo complexes are sufficient to elicit complex cargo trafficking behavior in living cells.

Several interesting points arise from our work. First, the short residence time of dynein that we observed is in contrast with the findings from previous research (McKenney et al., 2014; Schlager et al., 2014), which report a run length of up to 8 µm for dynein. In a recent preprint where single molecules of endogenously tagged dynein were visualized in neuron-inducible human stem cell lines, the authors found dynein to be capable of undertaking longer runs of ∼30 μm on an average (Fellows et al., 2023 *Preprint*). However, this run length represents those dyneins that were selected for their ability to undertake long-range movements (>10 μm) and was not representative of the entire population of dyneins in these neurons (which included those effectively diffusing on the MTs). While other in vitro results have reported dynein run lengths that are comparable with our results (King and Schroer, 2000; Ross et al., 2006), the dyneins in these studies might not have bound activating adaptors. It is worth noting that we observed minimal cargo diffusion upon MT depolymerization, indicative of the highly dense intracellular environment. In in vitro assays, the buffer density, as well as factors such as the molar excess of adaptors used, the ionic strength of the buffers, and the source and modifications on the MTs are all likely to differ from the in cellulo environment, likely explaining the disparity in dynein run length between our experiments and in vitro*.* Importantly, the run lengths and velocity of cargoes we measured in cellulo here are consistent with the well-established trafficking times of endocytic cargo delivery to lysosomes from multiple studies (Rink et al., 2005; Salova et al., 2017; Kornilova et al., 1996; Lakadamyali et al., 2006). The short, stop-and-go movements we observe are therefore sufficient for endosomal movement in the cell.

Second, from our study, it appears dynactin and endosomal cargoes remain either persistently bound to or remain in very close proximity to MTs. Since we did not have access to a BAC-based cell line that stably expressed fluorescent p150 (DCTN1) like the mDHC-GFP line used here, we resorted to using transient transfections and selecting cells for imaging that expressed low levels of GFP-p150. The persistent association of dynactin to MTs that we observed is in contrast to in vitro observations where recombinant human dynactin was not found to decorate pig brain–derived MTs (McKenney et al., 2014). Similarly, recombinant budding yeast dynactin was observed to have a weak interaction with axonemal MTs (Kardon et al., 2009). But when dynein was added to the mix, movement of dynactin was observed implying that dynactin could not independently bind to the MTs. However, in other instances (Culver-Hanlon et al., 2006), all p150 fragments containing the CAP-Gly domain bound to the MT. Dynactin that was overexpressed in cells was found to bind strongly to and bundle MTs (Quintyne and Schroer, 2002; Vaughan et al., 2002). In fact, dynactin–MT interactions have recently been shown to be important for increasing the on-rate of dynein onto MTs (Sanghavi et al., 2021). We propose that dynactin may assist in anchoring cargoes to the MT, allowing rapid initiation of movement upon dynein binding.

Third, since cytoplasmic dynein is the major minus end–directed motor that participates in endocytic membrane trafficking, how dynein interacts with and transports different types of cargo is an important question. Recent research suggests that cargo-specific adaptors like BicD2 (for Rab6-positive cargo) and Hook1/3 (for Rab5-positive cargo) have differential interaction with dynein (McKenney et al., 2014). These cargo adaptors could modulate the interaction time of dynein with the cargo, thereby leading to different kinetics. Such stochastic binding and unbinding would allow the same dynein molecule to sample and interact with a wide range of cargo. As EGF is highly likely to engage the EGFR, while dextran can simply be incorporated into endosomes without receptor binding, we speculate that the differences in EGF trafficking observed in our experiments are due to the engagement of the EGFR, leading to a higher number of motor adaptors being recruited to the endosome. We confirmed here that the motor adaptors Hook1 and Hook3 interact with EGF-containing endosomes, and at least Hook3 plays a role in EGF-containing endosome movement toward the cell center, consistent with previously published results (Xu et al., 2008). Hook1 and 3 have been demonstrated to persistently engage with cargo in vitro and in living cells via the FHIP proteins, where they affect dynein-mediated movement of Rab5-positive early endosomes (Christensen et al., 2021). Hook1 also mediates the long-range transport of early-endosome localized TrkB receptors in neurons (Olenick et al., 2019). Our observed colocalization of EGF-containing endosomes with Hook1 and 3, and disrupted localization of these endosomes upon Hook3 depletion is consistent with these motor adaptors being persistently associated with both endosomal cargoes and microtubules as observed by Christensen et al. (2021)*.* Together, our results suggest that two of the three members of the tripartite complex, dynactin and the motor adaptor, are persistently associated with cargo on the microtubule, whereupon stochastic interaction with diffusive dynein affects endosomal cargo movement to the cell center.

Fourth, while we only looked at degradation-targeted endosomal cargoes in this study, movement to the MT minus or plus ends (as during cargo recycling) could therefore also be tuned by slightly increasing the bias in one direction, for example by increasing the number of adaptors for dynein on a cargo destined toward the minus end.

Fifth, cellular morphology is likely to play a significant role in the kinetics of motor attachment and detachment from the MT. For instance, in highly polar, narrow axons of neuronal cells, reattachment of dynein to cargo localized close to the MT is likely to occur frequently due to the spatial confinement, as the reduced volume in which dynein could diffuse would lead to higher levels of interaction between the motor and cargo, and therefore more processive cargo trajectories.

Finally, we posit that a similar mechanism is at work in transporting plus end–directed cargo by kinesin motors (Redpath and Ananthanarayanan, 2023). More generally, both dyneins and kinesins can simultaneously affect cargo movement, even for those cargo that are transported toward the minus end on the average such as dextran and EGF.

Altogether, our study establishes that dynein acts in short spurts in a cellular environment, which are sufficient to drive endosomal transport from the cell periphery to perinuclear region within well-established time frames. Dynein achieves this by stochastic, transient binding to endosomal cargoes, and our experiments suggest cargo adaptor number and/or dynein diffusion upon cargo detachment may be central to determining the ultimate speed of cumulative cargo movement.

## Materials and methods

### Cell culture

WT HeLa cells (ATCC: CCL-2, RRID:CVCL_0030) were cultured in Dulbecco’s Modified Eagle Media (DMEM) containing 100 U/ml Penicillin, 0.1 mg/ml Streptomycin, 2 mM *L*-Glutamine (Cat# AT066; HiMedia Laboratories/Cat# 11965092; Thermo Fisher Scientific) and supplemented with 10% fetal bovine serum. DHC-GFP HeLa cells (Poser et al., 2008) were cultured in DMEM containing 100 U/ml Penicillin, 0.1 mg/ml Streptomycin, 2 mM *L*-Glutamine, and 400 µg/ml Geneticin (HiMedia Laboratories) and supplemented with 10% fetal bovine serum (Cat# F7524; Sigma-Aldrich/Moregate Biotech Fetal Bovine Serum—Sterile Filtered). Cells were grown in an incubator at 37°C under 5% CO_2_.

### Microscopy

Imaging was performed on a Nikon Ti2E inverted microscope equipped with a Toptica MLE laser combiner, Nikon H-TIRF Module, Yokogawa CSU-X1 spinning disk module, an Andor iXon 897 EMCCD camera, and an Oko Lab stage top incubator. The microscope was controlled using Nikon NIS Elements software or Micromanager (Edelstein et al., 2014). Alternately, a Zeiss ELYRA P1 with 2 Andor iXon+ EMCCD cameras controlled using Zen Black software (RRID:SCR_018163) or a Zeiss 800 Airyscan microscope (Zeiss Axio Observer Z1 laser scanning confocal microscope equipped with an Airyscan detector) controlled using Zen Blue software (RRID:SCR_013672), or a Nikon A1 resonant scanning confocal microscope equipped with GaAsP detectors controlled using NIS Elements (RRID:SCR_014329) was used. All live-cell microscopy was undertaken at 37°C in live-cell imaging solution (140 mM NaCl, 2.5 mM KCl, 1.8 mM CaCl2, 1 mM MgCl2, 4 mg/ml D-Glucose, 20 mM HEPES). All fixed cell imaging was undertaken at room temperature (23°C) in PBS.

### RNAi experiments

For all RNAi experiments, cells were first grown in glass-bottom imaging dishes (Cat #81518; Ibidi) for 48 h, transfected with the appropriate concentration of siRNA using Jet Prime transfection reagent (Cat #114; Polyplus), Lipofectamine 3000 (Cat #L3000001; Thermo Fisher Scientific), or Neon Electroporation (Thermo Fisher Scientific) and imaged after 48 h. For hDHC RNAi, the following siRNA sequence was used: 5′-ACA​UCA​ACA​UAG​ACA​UUC​A-3′. For p150 RNAi, the following siRNA sequences were used: 5′-GUA​CUU​CAC​UUG​UGA​UGA​A-3′, 5′-GAU​CGA​GAG​ACA​GUU​AUU​A-3′ (Watson and Stephens, 2006). siRNA oligonucleotides were procured from Eurogentec, Belgium, or SilencerSelect siRNA from Thermo Fisher Scientific (Hook1: s28011, Hook3: s228365). To quantify the knockdown of proteins, the cells were lysed 48 h after siRNA transfection, and SDS-PAGE and western blotting were performed with the cell lysates. The following primary antibodies were used to visualize proteins of interest and controls: Rabbit DYNC1H1 Polyclonal Antibody (Cat# PA5-49451, RRID:AB_2634905, 0.5 µg/ml; Thermo Fisher Scientific), Rabbit Dynactin 1 Polyclonal Antibody (Cat# PA5-37360, RRID:AB_2554027, 0.5 µg/ml; Thermo Fisher Scientific), Mouse GAPDH Loading Control Antibody (Cat# MA5-15738, RRID:AB_10977387, 0.5 µg/ml; Thermo Fisher Scientific), Rabbit Actin Polyclonal Antibody (Cat# ab8227, RRID:AB_2305186, 0.5 µg/ml; Abcam). The following secondary antibodies were used: Anti-Rabbit HRP (Cat# 31460, RRID:AB_228341, 0.16 µg/ml; Thermo Fisher Scientific) and Anti-Mouse HRP (Cat# 62-6520, RRID:AB_2533947, 0.3 µg/ml; Thermo Fisher Scientific) and Donkey Anti-Rabbit A555 (Cat# A32794, 0.05 µg/ml, RRID:AB_2762834; Thermo Fisher Scientific). Blots were imaged on a BioRad ChemiDoc Imaging System. Band intensity on blots was quantified using Fiji/ImageJ. Alternately, knockdown was quantified using immunofluorescence (Hook1 and Hook3) using Rabbit anti-Hook1 (Cat# ab151756, RRID:AB_3076228; Abcam), Rabbit anti-Hook3 (Cat# HPA024756, RRID:AB_1850913; Sigma-Aldrich), and Donkey Anti-Rabbit A647 (Cat# A32795, 0.2 µg/ml, RRID:AB_2762835; Thermo Fisher Scientific). 48 h after transfection with siRNA, cells were fixed with 4% PFA, transferred into PBS, and imaged using a Nikon A1 Scanning Confocal Microscope with 20× 0.75 NA air objective.

### Immunofluorescence

To immunostain p150 and tubulin, dynein and EGFR, and Hook1/3 and EGFR, cells were first grown for 48 h in glass-bottom imaging dishes (Cat# 81518; Ibidi) and fixed in ice-cold methanol at −20°C for 3 min. Cells were washed thrice in phosphate-buffered saline (PBS) for 5 min each and incubated in blocking buffer (5% BSA in PBS) for 60 min. Subsequently, the cells were incubated with p150 and α-tubulin antibodies in antibody dilution buffer (1% BSA in PBS) for 60 min. Cells were then washed thrice in PBS for 5 min and incubated with secondary antibodies in antibody dilution buffer for 45 min. At the end of incubation with secondary antibodies, the cells were washed with PBS and imaged immediately. The following primary antibodies were used: Rabbit DYNC1H1 Polyclonal Antibody (Cat# PA5-68173, RRID:AB_2691896, 1 µg/ml; Thermo Fisher Scientific), Rabbit Dynactin 1 Polyclonal Antibody (Cat# PA5-21289, RRID:AB_11155448, 2 µg/ml; Thermo Fisher Scientific), Mouse α Tubulin Monoclonal Antibody (Cat# 32-2500, RRID:AB_2533071, 2 µg/ml; Thermo Fisher Scientific), Rabbit anti-beta Tubulin antibody directly conjugated to AlexaFluor405 (Cat# ab179513, 250 µg/ml, RRID:AB_3073861; Abcam), Rabbit anti-Hook1 (Cat# ab151756, RRID:AB_3076228; Abcam), Rabbit anti-Hook3 (Cat# HPA024756, RRID:AB_1850913; Sigma-Aldrich), Mouse anti-EGFR (Cat# ab30, RRID:AB_303483; Abcam). The secondary antibodies used were Donkey Anti-Rabbit A555 Antibody (Cat# A-31572, RRID:AB_162543, 0.4 µg/ml; Thermo Fisher Scientific), Goat Anti-Mouse A647 Antibody (Cat# A28181, RRID:AB_2536165, 2 µg/ml; Thermo Fisher Scientific), Donkey Anti-Mouse A488 (Cat# A32766, RRID:AB_2762823, 0.2 µg/ml; Thermo Fisher Scientific), Donkey Anti-Mouse A405 (Cat# A48257, RRID:AB_2884884, 0.2 µg/ml; Thermo Fisher Scientific), Donkey Anti-Rabbit A555 (Cat# A32794, RRID:AB_2762834, 0.2 µg/ml; Thermo Fisher Scientific), and Donkey Anti-Rabbit A647 (Cat# A32795, RRID:AB_2762835, 0.2 µg/ml; Thermo Fisher Scientific). Where anti-β-tubulin was used in conjunction with another rabbit-derived antibody, the other antibody was first added for 2 h at room temperature, detected with an anti-rabbit secondary antibody, sample fixed in 2% PFA for 20 min at room temperature, and then anti-β-tubulin AlexaFluor405 was added for 1.5 h at room temperature.

To immunostain p150 and p62, p62 and α-tubulin, and EB1 and α-tubulin, the following method was used. Cells were first grown for 48 h in glass-bottom imaging dishes and fixed in ice-cold methanol at −20°C for 3 min. Subsequently, the cells were washed thrice in PBS for 5 min each and incubated in antibody dilution buffer (2% BSA, 0.1% Triton X-100, in PBS) for 10 min. Next, the cells were incubated in antibody dilution buffer containing the appropriate antibodies for 60 min. Cells were washed thrice in PBS for 5 min each and incubated with appropriate secondary antibodies in antibody dilution buffer for 30 min. At the end of incubation with secondary antibodies, the cells were washed well in PBS and imaged immediately. The following primary antibodies were used: Rabbit Dynactin 1 Polyclonal Antibody (Cat# PA5-21289, RRID:AB_11155448, 2.5 µg/ml; Thermo Fisher Scientific), Rabbit Alpha Tubulin Monoclonal Antibody (Cat# PA5-22060, RRID:AB_11154084, 2 µg/ml; Thermo Fisher Scientific), Mouse Dynactin 4 Monoclonal Antibody (Cat# MA5-17065, RRID:AB_2538536, 2 µg/ml; Thermo Fisher Scientific), and Mouse EB1 Monoclonal Antibody (Cat# 41-2100, RRID:AB_2533500, 2 µg/ml; Thermo Fisher Scientific). The secondary antibodies used were Donkey Anti-Rabbit A647 Antibody (Cat# A32795, RRID:AB_2762835, 2 µg/ml; Thermo Fisher Scientific) and Goat Anti-Mouse A555 Antibody (Cat# A-21422, RRID:AB_2535844, 2 µg/ml; Thermo Fisher Scientific).

To immunostain Rab5 and p62, cells were first fixed in 4% PFA for 15 min. The cells were washed well in PBS and incubated for 45 min in antibody staining solution (0.2% saponin, 0.1% BSA, and 0.02% sodium azide in PBS) containing primary antibodies against Rab5 and p62. Subsequently, cells were washed well in PBS and incubated for 45 min in antibody-staining solution containing appropriate secondary antibodies. Cells were washed well in PBS and imaged immediately. The following primary antibodies were used: Rabbit Rab5 Monoclonal Antibody (Cat# 3547, RRID:AB_2300649, 14 µg/ml; Cell Signaling Technology) and Mouse Dynactin 4 Monoclonal Antibody (Cat# MA5-17065, RRID:AB_2538536, 2 µg/ml; Thermo Fisher Scientific). The secondary antibodies used were Donkey Anti-Rabbit A647 Antibody (Cat# A32795, RRID:AB_2762835, 2 µg/ml; Thermo Fisher Scientific) and Goat Anti-Mouse A555 Antibody (Cat# A-21422, RRID:AB_2535844, 2 µg/ml; Thermo Fisher Scientific).

Immunostained samples were imaged using a Zeiss 800 Airyscan microscope using a 63× 1.4 NA oil immersion lens with a 42.5 nm pixel size.

### Dextran and EGF uptake

To visualize fluorescent dextran vesicles, HeLa cells that were grown in glass-bottom imaging dishes (Cat# 81518; Ibidi) for 48 h were transferred to serum-free DMEM for 4 h in a 37°C CO_2_ incubator. Subsequently, the cells were pulsed for 10 min in complete DMEM containing 200 µg/ml A647-Dextran (D-22914; Invitrogen). Cells were then washed well with live-cell imaging solution before proceeding for microscopy. In all experiments, imaging was completed within 45 min of dextran uptake.

To visualize fluorescent EGF vesicles, HeLa cells that were grown in glass-bottom imaging dishes (Cat# P35G-1.5-14-C; Mattek) for 24 h were transferred to serum-free DMEM for 1 h in a 37°C CO_2_ incubator. Cells were washed in serum-free DMEM, then incubated with 1 nM EGF (Cat# E9644; Sigma-Aldrich) labeled with Alexa647 (Cat# ab269823; Abcam) in phenol-free, serum-free DMEM for a minimum of 8 min prior to imaging. In all experiments, cells were imaged between 8 and 20 min of EGF uptake.

### Transfection

To visualize fluorescently tagged proteins, cells were transfected with the appropriate plasmids using Jet Prime Transfection Reagent (Cat# 114; Polyplus) or Lipofectamine 3000 (Cat# L3000001; Thermo Fisher Scientific). 3 h after transfection, the cells were washed well in PBS, grown in complete DMEM at 37°C, and imaged ∼20 h later. The following plasmids were used in this study: (i) mCherry-tubulin was a gift from Mariola Chacon, Technische Universität Dresden, Dresden, Germany; (ii) Gal4T-mCherry was a gift from Thomas Pucadyil, Indian Institute of Science Education and Research Pune, India; (iii) mCherry-DCTN1 was a gift from Kozo Tanaka, Tohoku University, Sendai, Japan; (iv) mCherry-Rab5 was a gift from Gia Voeltz (plasmid 49201; RRID:Addgene_49201; Addgene); (v) EGFP-Rab7A (in the pEGFP-C1 backbone) was a gift from Dr. Neftali Flores-Rodriguez (Sydney Microscopy & Microanalysis, University of Sydney, Sydney, Australia); (vi) LAMP1-mGFP was a gift from Esteban Dell’Angelica (plasmid 34831; RRID:Addgene_34831; Addgene); (vii) pEGFP-p150 was a gift from David Stephens (plasmid 36154; RRID:Addgene_36154; Addgene).

### HILO microscopy and particle tracking

For HILO microscopy, a Nikon 100× 1.49 NA TIRF objective was used. We optimized the HILO microscopy setting for each cell as described previously (Tirumala and Ananthanarayanan, 2023). Briefly, first, to avoid overexpression artifacts, we visualized cells expressing low levels of mDHC-GFP. The diameter of the illuminated area was kept constant at 30 µm and the long axis of the cell was aligned perpendicular to the excitation laser. Finally, we adjusted the angle of incidence of the excitation laser, such that the fluorescent spots in a plane ∼0.5 µm from the coverslip appeared bright and distinct. The incidence angle and orientation of the excitation laser beam were adjusted using the H-TIRF module and the laser power (50 mW at fiber) was kept at 40%. To visualize single molecules of dynein in Fig. 1, Fig. S1 e and f, and Figs. S2 and S4, time-lapse images were acquired at 50 frames per s (fps) with 20-ms exposure per frame for a total of 10 s per video.

To improve the signal-to-noise ratio, a five-frame sliding average of the time-lapse images was used for particle tracking. In these videos, the appearance and subsequent disappearance of intensity at a particular location was classified as a binding event. Such binding events were visually identified and tracked from the start to end frame using Low Light Tracking tool (Krull et al., 2014) in Fiji/ImageJ (RRID:SCR_002285; Schindelin et al., 2012). The fluorescent spots were classified as stationary, minus end moving, or plus end moving by marking a region close to the nucleus as the minus end and calculating the displacement of fluorescent spots with respect to the minus end. Particles moving away from the nucleus for >0.32 µm were classified as plus end moving, particles with displacement >0.32 µm toward the nucleus were minus end directed, and particles with net displacement <0.32 µm were classified as stationary. These displacements refer to the total displacement of the dynein particle for the entire duration for which it was tracked. The 0.32 µm threshold was arrived at after multiplying the 80 nm error in particle tracking with four frames, ensuring that any displacement that is an artifact of the tracking error is discounted in our analysis.

For dual channel imaging of dynein and cargo, videos were acquired at 20 fps with 25-ms exposure per channel for dynein and dextran (in cells depleted of hDHC using RNAi), and at 6 fps with 33-ms exposure per channel for dynein and EGF. To observe dynein–dextran interaction with a better signal-to-noise ratio, a two-frame sliding average of time-lapse images was used.

For 100-s dextran and EGF imaging experiments, an alpha Plan-Apochromat 100× 1.46 NA Oil Elyra TIRF objective was used. Consistent HILO settings were used across all long-term dextran and EGF uptake experiments. Fluorophores were excited using a 642 nm laser (150 mW at source) at a TIRF angle of 63°. Exposure times of 50 ms with a frame interval of 100 ms were used (total frame time, 125 ms with frame transfer), and imaging was undertaken for 1,000 frames (125 s in total). The movement of dextran vesicles was classified in the following way: the movement between two consecutive frames was classified as zero if it was <20 nm (the tracking accuracy of low light tracking tool under the imaging conditions used [Krull et al., 2014]). Then, displacement for four consecutive frames toward or away from the nucleus was classified as a minus end– or plus end–directed run, and a zero displacement for two or more consecutive frames was classified as a pause.

### Spinning disk confocal microscopy, Airyscan microscopy, and image analysis

To quantify the correlation between expression level of dynein and its clustering, z-stack images of live HeLa cells expressing mDHC-GFP were acquired using a 60×, 1.4 NA objective with 50-ms exposure. The intensity comparison was done for the lowest plane at which clusters were distinctly visible. The dynamics of mCherry-p150 in cells were visualized by acquiring 60-s-long movies of the cell with a 1.4 NA 60× objective, 100-ms exposure per frame, and 1-s interval between frames. The dynamics of dextran vesicles along the MTs was visualized by acquiring 30-s-long movies of the cell with a 60×, 1.4 NA objective, 100-ms sequential exposure/channel, and 1-s interval between frames. The spinning disk+SRRF images were obtained by taking the mean of the radiality map of 100 images of a single field acquired under a 100×, 1.49 NA objective with the spinning disk confocal microscopy setup. The radiality magnification in SRRF was set to 4 for all experiments. To visualize the association between MTs and dextran vesicles in live cells, the SRRF acquisition settings used were 20-ms exposure, ring radius of 0.5 for MTs, and 3 for dextran. To visualize p62 with tubulin and EB1 with tubulin, 20-ms exposure and a ring radius of 0.5 was used. To visualize p150 with tubulin and p150 with p62, the SRRF acquisition settings used were 100-ms exposure and ring radius of 1. Finally, to visualize p62 with Rab5, 20-ms exposure was used in both channels. A ring radius of 0.5 and 3 was used for p62 and Rab5, respectively.

Kymographs for all live-cell images are whole-cell kymographs generated in one of two ways (second way indicated in parantheses) as follows: first, the cell was oriented so that its long axis was vertical (horizontal) within the x-y plane of the image window. The “Reslice” function in Fiji/ImageJ was applied, starting left (top) and rotating 90° (option not selected). Then a maximum intensity projection was obtained to selectively visualize trajectories along the long axis.

The co-occurrence of signals with each other (e.g., p150 with p62) was quantified using object-based colocalization analysis (Bolte and Cordelières, 2006) from SRRF or Airyscan images. For example, for p150 and p62, SRRF images of p150 were first thresholded for the top 5% of intensity or auto-thresholded using the default “IsoData algorithm” in Fiji. The number of p150 spots was counted as *Np*150 and a mask was created. Then, the SRRF image of p62 in the same cells was combined with the p150 mask using the “AND” operation in Fiji. The resultant image was thresholded and the number of spots was counted (*N*p62). *N*p62/*N*p150*100 gave the percentage of p150 spots with p62. A similar procedure was used to quantify the percentage of co-occurrence of all other signals from both SRRF and Airyscan images. To quantify the effect of p150 knockdown on the levels of p150, p62 along the MT, regions of interest (ROIs) were drawn along distinct MT segments that were randomly chosen in SRRF images of MT, and the mean intensities of p150 or p62 in these ROIs were measured.

Endosome mean dispersion and displacement from the centroid were quantified following manual segmentation of the cell and identical thresholds for every image to segregate endosomal signal from the background using a Fiji plugin developed by Dr. Michael Carnell at the Katarina Gaus Light Microscopy Facility at University of New South Wales (Redpath et al., 2019).

### CLEM

**Figure S7.**
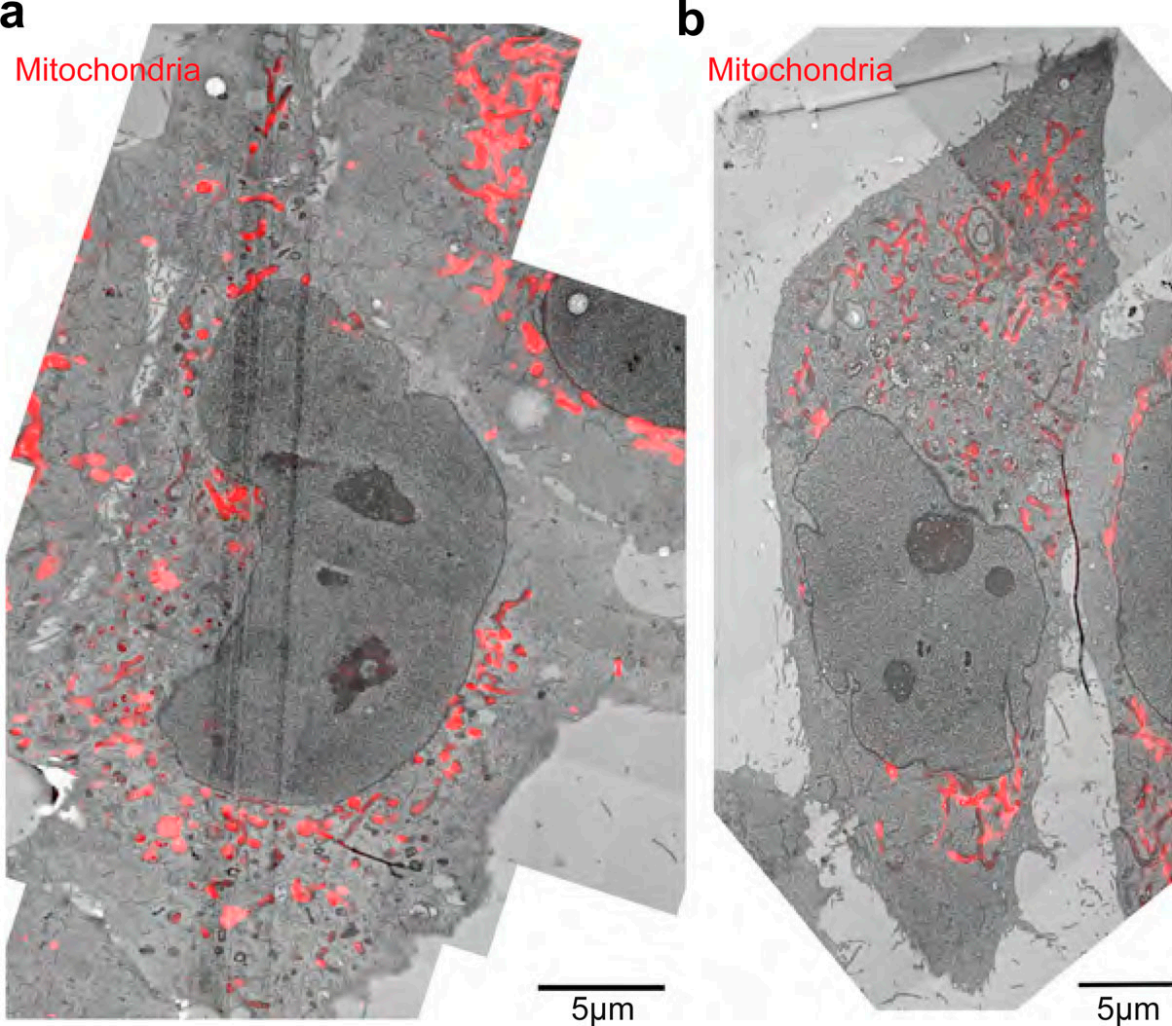
**Correlation and re-registration in the z-dimension. (a)** Dextran-labeled HeLa cell from Fig. 3, d–f, with the overlay of the MitoTracker Orange signal showing accurate fitting in z between TEM and 3D confocal microscopy. **(b)** EGF-labeled HeLa cell from Fig. 3, g–i, with the overlay of the MitoTracker Orange signal showing accurate fitting in the z-dimension between TEM and 3D confocal microscopy.

HeLa cells were grown for 24 h on gridded glass-bottom imaging dishes (Cat #P35G-1.5-14-CGRD; Mattek), following which cells were transfected with EGFP-tubulin (Cat #56450; AddGene) overnight. Cells were transferred into serum-free, phenol-free DMEM for 1 h, following which A647-dextran or EGF-647 was added to cells for 10 min. Cells were extensively washed in PBS and fixed in 4% PFA containing 0.2% glutaraldehyde (Cat #354400; Sigma-Aldrich) for 1 h at room temperature. Following fixation, cells were dyed with MitoTracker Orange CMTMRos (Cat #M7510) to fluorescently label mitochondria through the cell volume and then imaged on a Zeiss LSM 800 with Airyscan detector using a 63× 1.4 NA oil immersion lens, with a pixel size of 42.5 nm and z-slice spacing of 150 nm. The MitoTracker-stained mitochondria served as markers for correlation and registration of the relevant confocal z-slice with that of the corresponding transmission electron microscope (TEM) z-slice based on the identification of mitochondrial morphology and distribution at each slice (Fig. S7). After imaging, cells were further fixed in 2.5% glutaraldehyde in 0.1 M sodium cacodylate for 1 h at room temperature. All the subsequent processing steps were carried out in a BioWave microwave (Pelco). After fixation, samples were washed with cacodylate buffer, additionally fixed in reduced osmium (1% osmium tetroxide-1.5% potassium ferricyanide) solution followed by buffer wash, and resuspended in 2% aqueous OsO_4_ (osmium tetroxide; ProScitech). Washed samples were then stained with 2% wt/vol Phosphotungstic acid in 30% ethanol at 60° for 1 h, followed by a 30% ethanol wash at room temperature. Samples were then stained with 2% aqueous uranyl acetate prepared in 30% ethanol and incubated at 4°C for 1 h. Serial dehydration was then continued at this point in increasing percentages of ethanol, following which cells were serially infiltrated with Durcupan ACM (Cat #44610; Sigma-Aldrich). Fresh 100% resin was then added and polymerized at 60°C overnight. Ultrathin sections were cut on an ultramicrotome (UC6: Leica) and imaged at 100 kV on a JEOL1400 TEM fitted with a Phurona EMSIS CMOS camera operated using Digital Micrograph software, using a 5 × 5 stitching matrix. Light and electron microscopy images were subsequently overlayed and correlated using the Correlia plugin for Fiji/ImageJ (Rohde et al., 2020) and briefly described here. Using the signal from the orange fluorescent mitochondrial dye (Fig. S7), a TEM section of the cell 65 nm thick was correlated with the best fit confocal z-slice within the realms of the confocal slice thickness of 150 nm. An overlay of the TEM image and corresponding confocal image showing the mitochondrial signal only at the relevant z-slice was created for cells that had taken up both A647-dextran and A647-EGF. Once the overlay confirmed the right cell volume, then the green signal from EGFP-tubulin and the red signal from A647-dextran or EGF-647 were overlayed onto the same TEM image, and this enabled visualization of the proximity of dextran and EGF endosomes with respect to MTs. The distance of the dextran- and EGF-containing endosomes from the MTs was estimated by measuring the straight-line distance between the endosome membrane and the closest MT.

### Curve fitting and data visualization

The intensity fits in Fig. 1 c were performed as described previously (Tirumala and Ananthanarayanan, 2023; Ananthanarayanan et al., 2013; Ananthanarayanan and Tolić, 2015). Briefly, the intensity values of tracked dynein molecules were plotted as a histogram. This distribution was fit by a sum of two Gaussians with the same standard deviation (*σ*). The smaller mean was constrained between 15 and 30 and the larger mean was constrained between 35 and 50. Fitting with a sum of two Gaussian distributions yielded better goodness of fit than fitting with a log-normal distribution (R^2^ of 0.99 versus 0.96, respectively).

For the exponential fit in Fig. 1 f, the probability versus residence time (*τ*) histogram of dynein on the MTs was obtained from the lengths of all single molecule binding events. From this *P* (*τ*) = 1 − cumulative frequency of the residence time was obtained and was fit to *λe*^*−λt*^ to obtain *λ*, the off-rate from MTs. The average residence time was given by 1*/λ*.

All data was plotted using MATLAB (RRID:SCR_001622; MathWorks) or GraphPad Prism (RRID:SCR_002798). Figures panels were prepared using Adobe Illustrator (RRID:SCR_010279).

### Statistical analysis

Statistical analyses were performed in Matlab. Data were tested for normality using kstest2 or chi2gof in Matlab before opting for parametric or non-parametric tests. The specific tests performed are indicated in the respective figure captions. Box plots show the median, lower and upper quartiles, outliers, and the minimum and maximum values that are not outliers. The statistical tests or SD calculations were performed with the number of data points referring to individual cells, tracks, or endosomes (*n*) pooled over multiple independent experiments.

### Online supplemental material

Fig. S1 shows the visualization of single molecules of dynein using HILO microscopy. Fig. S2 shows mDHC-GFP is functional in HeLa cells depleted of endogenous DHC. Fig. S3 shows dynactin’s association with MTs. Fig. S4 shows dextran as an endosomal cargo marker and EGFR’s interaction with Hook proteins. Fig. S5 shows dextran and EGFR’s interaction with dynein. Fig. S6 shows the net movement of dextran and EGF endosomes over time. Fig. S7 shows correlation and re-registration in the z-dimension. Video 1 shows dynein binding events. Video 2 shows the dynamics of p150. Video 3 shows dextran is in Rab5^+^ compartments. Video 4 shows cargo–MT interaction. Video 5 shows intracellular crowding prevents cargo diffusion. Video 6 shows the movement of dextran vesicles in HeLa cells. Video 7 shows the movement of EGF vesicles in HeLa cells. Video 8 shows the activation of dynein upon dextran vesicle binding. Video 9 shows the activation of dynein upon EGF vesicle binding. Video 10 shows EGF endosomes undertake large movements toward the nucleus.

## Supplementary Material

SourceData F3 is the source file for Fig. 3.

SourceData FS2 is the source file for Fig. S2.

SourceData FS3 is the source file for Fig. S3.

SourceData FS5 is the source file for Fig. S5.

## Data Availability

All data and analysis routines are available from the corresponding author (V. Ananthanarayanan: vaish@unsw.edu.au) upon reasonable request.
