# Supplementary figures and images for: Single-molecule imaging of stochastic interactions that drive dynein activation and cargo movement in cells
Source: J Cell Biol. 2024 Jan 19;223(3):e202210026. doi: 10.1083/jcb.202210026 (PMC10798859; doi:10.1083/jcb.202210026)

**f**

p150

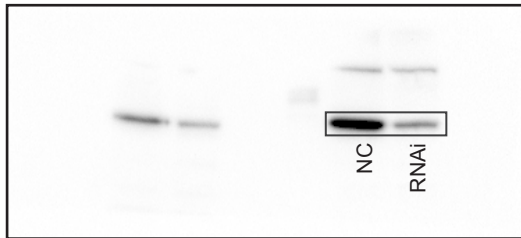

ladder

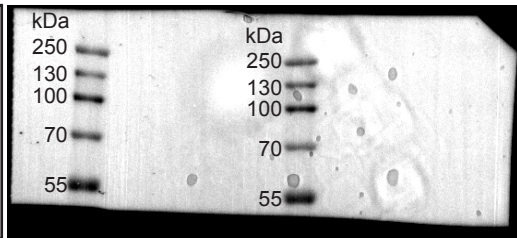

Actin

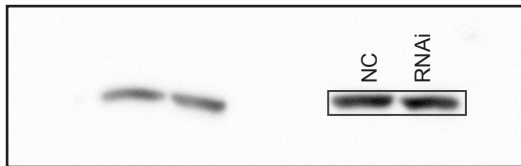

ladder

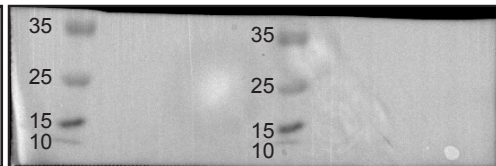

kDa

kDa

Supplement: SourceData F3 — is the source file for Fig. 3. [file JCB_202210026_SourceDataF3.pdf]

**b**

DHC

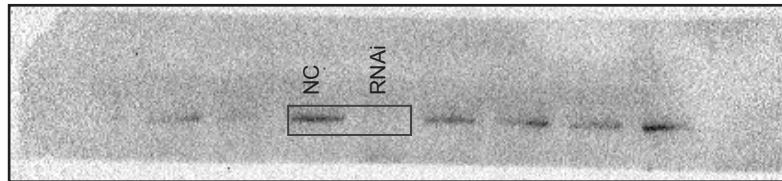

GAPDH

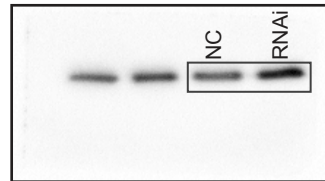**e**

DHC

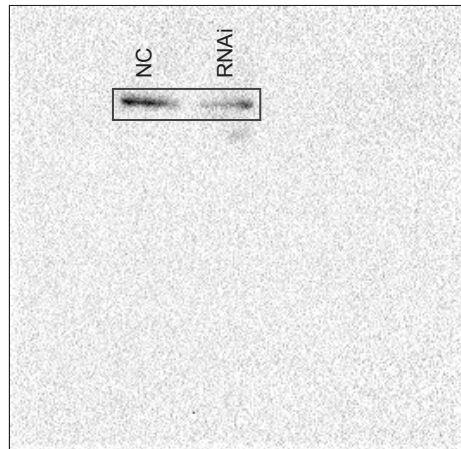

p150

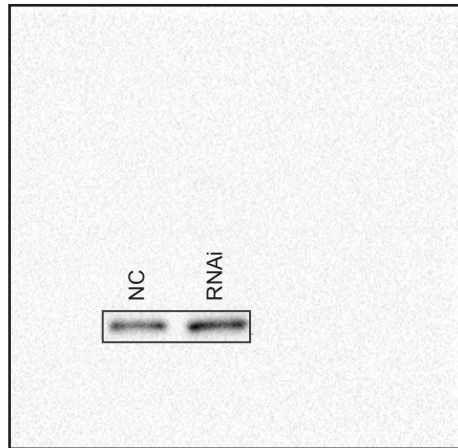

ladder

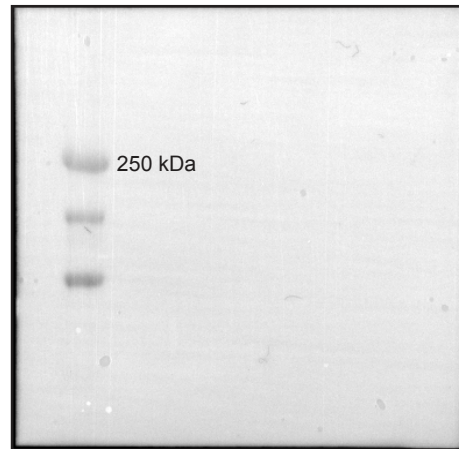

Supplement: SourceData FS2 — is the source file for Fig. S2. [file JCB_202210026_SourceDataFS2.pdf]

g

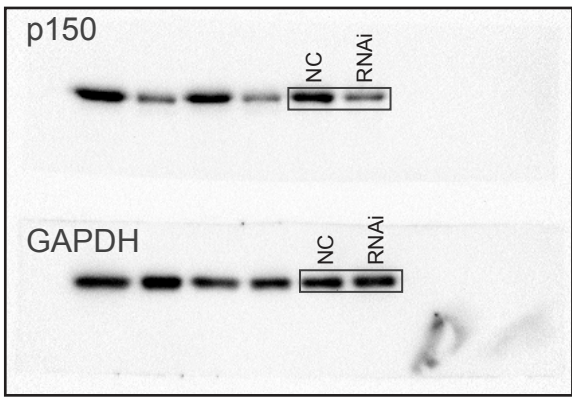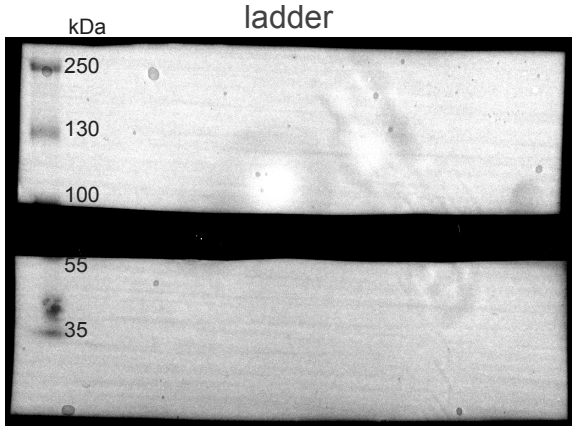

j

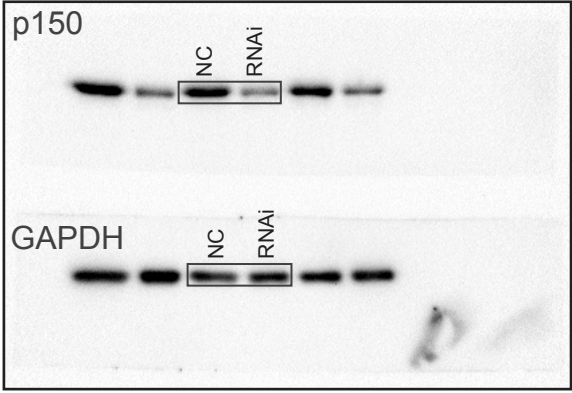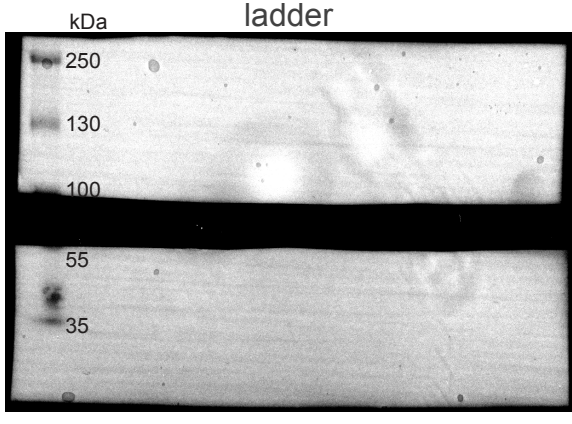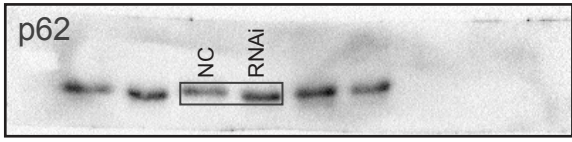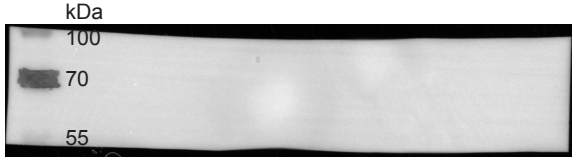

i

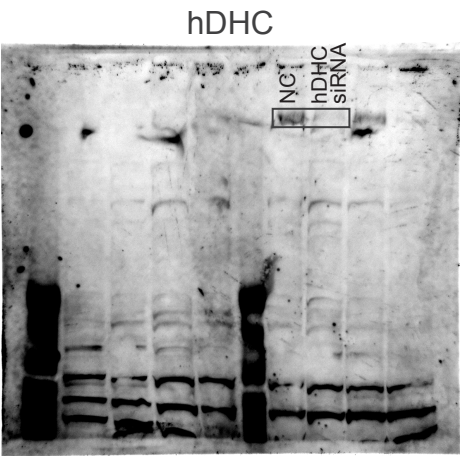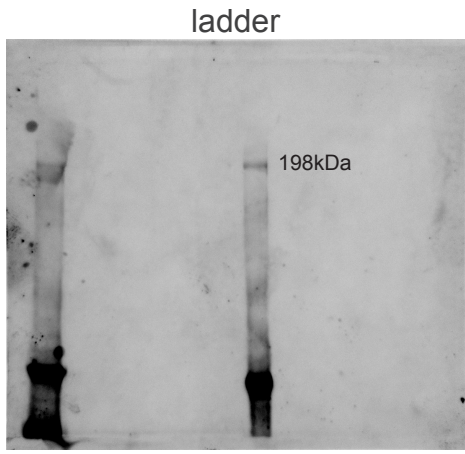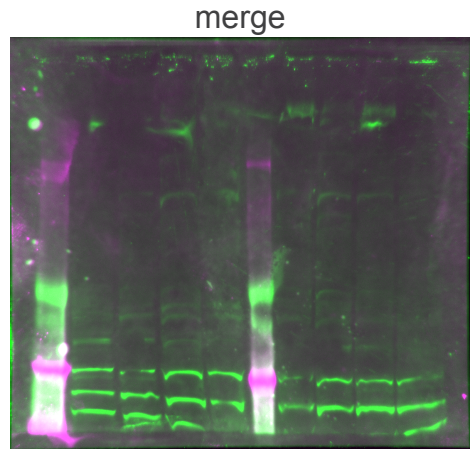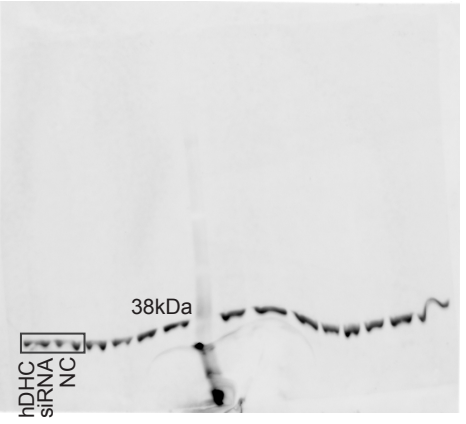

Supplement: SourceData FS3 — is the source file for Fig. S3. [file JCB_202210026_SourceDataFS3.pdf]

**e**

DHC

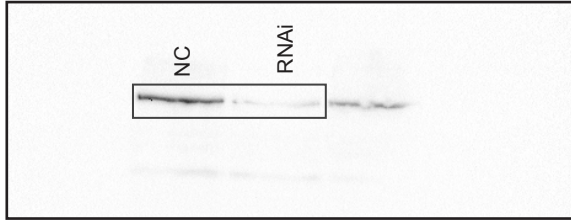

GAPDH

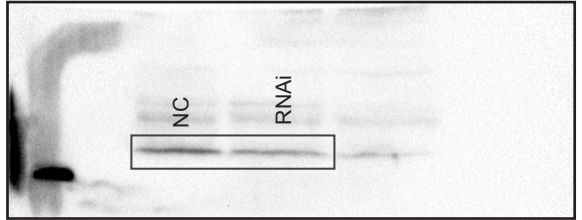

Supplement: SourceData FS5 — is the source file for Fig. S5. [file JCB_202210026_SourceDataFS5.pdf]
